# Spermidine improves gut barrier integrity and gut microbiota function in diet-induced obese mice

**DOI:** 10.1080/19490976.2020.1832857

**Published:** 2020-11-05

**Authors:** Lingyan Ma, Yinhua Ni, Zhe Wang, Wenqing Tu, Liyang Ni, Fen Zhuge, Aqian Zheng, Luting Hu, Yufeng Zhao, Liujie Zheng, Zhengwei Fu

**Affiliations:** a College of Biotechnology and Bioengineering, Zhejiang University of Technology, Hangzhou, China; b Research Institute of Poyang Lake, Jiangxi Academy of Sciences, Nanchang, China.; c Institute of Translational Medicine, the Affiliated Hospital of Hangzhou Normal University, Hangzhou, Zhejiang, China

**Keywords:** Spermidine, intestinal barrier function, gut microbiota, insulin resistance, short-chain fatty acid

## Abstract

Obesity is associated with impaired intestinal barrier function and dysbiosis of the gut microbiota. Spermidine, a polyamine that acts as an autophagy inducer, has important benefits in patients with aging-associated diseases and metabolic dysfunction. However, the mechanism of spermidine on obesity remains unclear. Here, we show that spermidine intake is negatively correlated with obesity in both humans and mice. Spermidine supplementation causes a significant loss of weight and improves insulin resistance in diet-induced obese (DIO) mice. These effects are associated with the alleviation of metabolic endotoxemia and enhancement of intestinal barrier function, which might be mediated through autophagy pathway and TLR4-mediated microbial signaling transduction. Moreover, spermidine causes the significant alteration of microbiota composition and function. Microbiota depletion compromises function, while transplantation of spermidine-altered microbiota confers protection against obesity. These changes might partly be driven by an SCFA-producing bacterium, *Lachnospiraceae NK4A136 group*, which was decreased in obese subjects and subsequently increased by spermidine. Notably, the change of *Lachnospiraceae NK4A136 group* is significantly correlated with enhanced gut barrier function induced by spermidine. Our results indicate that spermidine supplementation may serve as a viable therapy for obesity.

## Introduction

The prevalence of obesity has increased worldwide in the past ~50 years and has reached pandemic proportions.^1^ The pathophysiology of obesity is multifactorial and linked with imbalanced energy metabolism, and it is influenced by complex interactions between genetic, epigenetic, dietary, lifestyle, and environmental factors.^2^ Obesity greatly increases the incidence rates of chronic diseases, including cancer.^1,3^ Therefore, scientifically proven ways to enhance weight loss are urgently needed.

Intestinal barrier dysfunction and the subsequent translocation of bacteria and bacterial products are now considered to be important mechanisms associated with obesity.^4,5^ Impaired barrier function could result in enhanced intestinal permeability and thereby facilitate the translocation of microbiota-derived endotoxins, such as lipopolysaccharide (LPS) into the systemic circulation.^6^ The influx of the immune-stimulatory microbial ligand LPS is detected by Toll-like receptor 4 (TLR4), which initiates proinflammatory cascade signaling that leads to the promotion of insulin resistance.^7^ Thus, novel therapeutic approaches or agents that target intestinal barrier integrity might be promising strategies for the prevention and treatment of obesity.

On the other hand, the gut microbiota is critical for the interaction between the host and diet, which modulates the physiological function of the host.^8^ Accumulating evidence has shed light on an entirely new perspective suggesting that the gut microbiota is a driving force in the pathogenesis of obesity and metabolic syndrome.^9,10^ In addition, pivotal metabolites produced by the microbiota, such as short-chain fatty acids (SCFAs), have a beneficial effect on the prevention and treatment of metabolic syndrome.^11,12^ In particular, butyrate was demonstrated to attenuate endotoxemia and protect intestinal barrier function.^11,13^ Therefore, potential therapies, such as dietary modification, fecal microbiota transplant (FMT) and probiotic or prebiotic interventions, have been widely investigated for their effects on reestablishing gut homeostasis and improving insulin resistance by manipulating the composition and function of the gut microbiota.^14,15^

Spermidine, a natural polyamine, can either be obtained orally from exogenous dietary sources or be produced by commensal intestinal bacteria and cellular biosynthesis.^16^ The concentration of spermidine declines in an age-dependent manner and epidemiological studies have revealed that the increased dietary uptake of spermidine reduces overall mortality associated with cardiovascular diseases and cancer.^17^ The beneficial effects exerted by spermidine on aging and various diseases are mainly attributed to its role as a physiological autophagy inducer in a variety of model organisms.^16,18^ Recent studies have demonstrated that spermidine treatment protects against liver fibrosis and hepatocarcinogenesis by activating the microtubule-associated protein 1S (MAP1S)-mediated autophagy pathway.^19,20^ Moreover, spermidine administration attenuated weight gain and the comorbidities of obesity induced by hypercaloric pro-diabetic regimens, and this effect was correlated with autophagy activity in white adipose tissue (WAT).^21^ However, it is still not clear whether spermidine could protect gut barrier function by inducing autophagy, and how spermidine modifies the gut microbiota and contributes to metabolic improvement remains unresolved.

In the present study, we hypothesized that the administration of spermidine would exert protective effects on diet-induced obesity (DIO) in mice by both autophagy-dependent and autophagy-independent pathways. The impact and mechanism of spermidine on insulin resistance, inflammation, and the gut barrier and gut microbiota in DIO mice will be investigated. The results will inform scientific solutions for the treatment of obesity, type 2 diabetes, and metabolic syndromes by exogenous spermidine supplementation.

## Results

### Spermidine intake is negatively correlated with the obesity index in both humans and mice

**Figure 1.**
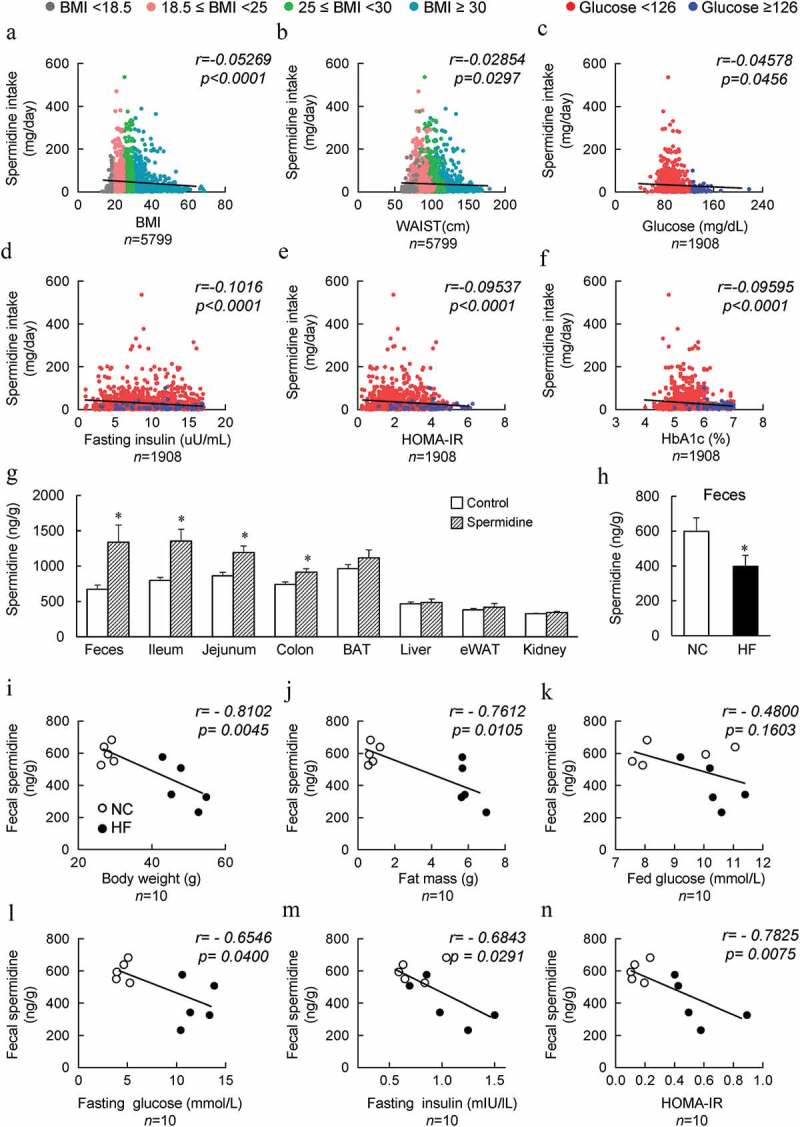
**Spermidine intake is negatively correlated with the obesity index in both humans and mice**. (a-f) Correlations of the individual daily spermidine intake with body mass index (BMI) (a), WAIST (b), fasting glucose (c), fasting insulin (d), homeostatic model assessment-insulin resistance (HOMA-IR) (e), and hemoglobin A1c (HbA1c) (f). (g) Spermidine distribution in mice (*n* = 6). (h) Concentration of spermidine in the feces of untreated NC- and HF- fed mice (*n* = 6). (i-n) Correlations of spermidine untreated NC- and HF- fed (*n* = 5) mice fecal spermidine concentrations with mice body weight (i), fat mass (j), fed glucose (k), fasting glucose (l), fasting insulin (m) and HOMA-IR (n) Values for *r* and *p* are indicated in each graph

The clinical characteristics of healthy and obese individuals without diabetic conditions were collected from the National Health and Nutrition Examination Survey (NHANES). Daily spermidine intake was calculated from the NHANES dietary data and nutrient database for polyamine intake. Pearson correlation was performed to assess the relationship between spermidine intake and the parameters associated with obesity. Daily intake of spermidine was negatively correlated with BMI, waist circumference and the homeostatic model assessment of insulin resistance (HOMA-IR) index and fasting serum glucose, fasting insulin, and HbA1c levels (Figure 1a-f). Moreover, as BMI and WASTE increases, daily spermidine intake decreased (Figure 1a and 1b). Importantly, the high glucose, insulin, HOMA-IR and HbA1c are generally associated with lowered spermidine intake (Figure 1c-f). These results suggested that the presence of a negative correlation between spermidine intake and obesity-associated parameters. Next, the tissue distribution of spermidine was determined after exogenous spermidine supplementation, and increased levels of spermidine were mainly found in the gut and feces (Figure 1g). In addition, the level of spermidine in the feces was decreased significantly in high-fat diet (HF)-induced DIO mice compared to normal chow (NC) fed mice (Figure 1h). Similar to the findings in humans, spermidine levels in feces from untreated NC-fed lean and HF-fed obese mice were found to be negatively correlated with body weight, fat mass, serum glucose levels both in the fed and fasting states, fasting serum insulin levels, and the HOMA-IR index (Figure 1i-n). Therefore, spermidine intake might be one potential factor that contributes to the prevalence of obesity.

### Spermidine administration ameliorates HF diet-induced metabolic syndrome

**Figure 2.**
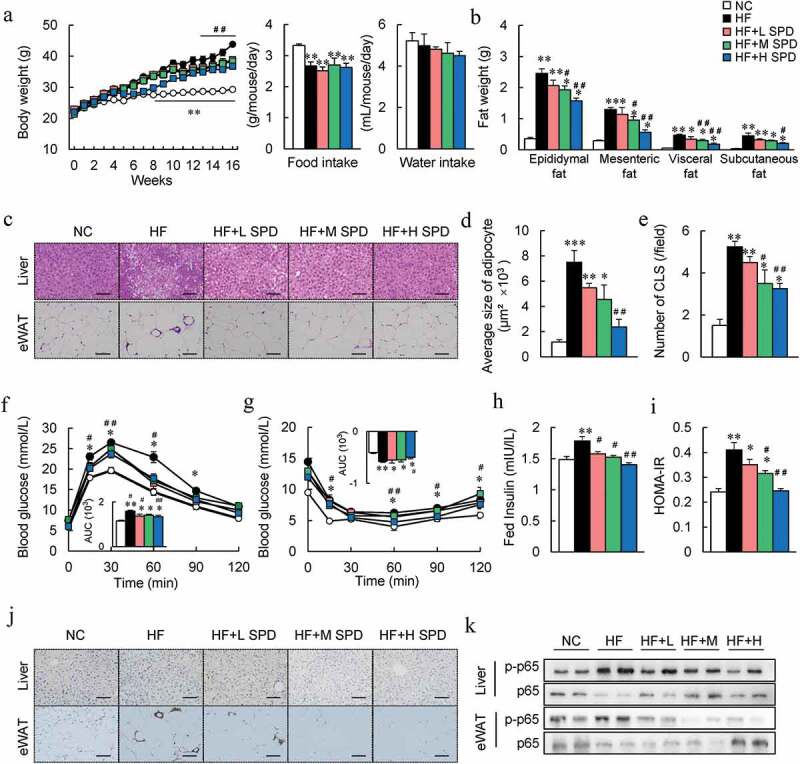
**Spermidine administration ameliorates HF diet-induced metabolic syndrome**. (a) Mice body weight, food and water intake (*n* = 8). (b) Adipose tissue weight (*n* = 8). (c) Representative H&E-stained sections from liver and eWAT. Scale bar: 100 µm. (d) Average size of adipocyte. (e) Number of crown-like structure (CLS) in each field. (f) Glucose tolerance tests (GTTs). AUC, area under the curve (*n* = 6). (g) Insulin tolerance tests (ITTs) (*n* = 6). (h) Plasma fed insulin levels (*n* = 6). (i) HOMA-IR index (*n* = 6). (j) Immunohistochemistry staining for F4/80 in the liver and eWAT, Scale bar: 100 µm. (k) Immunoblots of *p*-NF-κB p65 levels in the liver and eWAT (*n* = 5). Data were presented as the means ± SEM, *p < .05, ** *p* < .01 vs. NC; ^#^ *p* < .05, ^##^ *p* < .01 vs. HF

To assess the effects of spermidine on HFD-induced obesity in mice, spermidine was administered to DIO mice via their drinking water. After 16 weeks of administration, spermidine intake reduced diet-induced body weight gain in a dose-dependent manner without affecting food and water intake compared to HF controls (Figure 2a and FigureS1A). The reduction in weight gain in spermidine-fed mice was largely attributed to a significant decrease in fat mass rather than muscle weight (Figure 2b and Fig. S2B). This was also confirmed by the size of adipocytes in epididymal white adipose tissue (eWAT) (Figure 2c, d). Although the liver weight was not affected by spermidine, histological analysis and hepatic lipid content revealed higher lipid accumulation in the livers of DIO mice, which was decreased by spermidine (Figure 2c, Fig. S1C and S1D).

Moreover, HF-induced glucose intolerance, insulin resistance, and hyperinsulinemia were also improved by spermidine (figure 2f-i). These results were associated with the enhancement of insulin signaling in the liver and eWAT of spermidine-treated mice (Fig. S1E and S1F). F4/80 immunostaining revealed increases in F4/80^+^ macrophages in the liver as well as in the crown-like structure in eWAT of DIO mice, and spermidine administration reduced the activation of macrophages (Figure 2e, j). Furthermore, spermidine attenuated the inflammatory responses in the liver and eWAT of DIO mice, characterized by the phosphorylation of *p*-p65 NF-κB (Figure 2k and Fig. S1G). Furthermore, although exogenous spermidine intake failed to increase spermidine in adipose tissue and liver (Figure 1g), but significantly upregulated the expressions of *Odc, Srm*, and *Sms*, the key enzyme genes involved in the polyamines metabolism (Fig. S1H).

The BAT weight, as well as lipid accumulation increased significantly in DIO mice, which were decreased by spermidine (Fig. S2A and S2B). Spermidine treatment increased the core body temperature of DIO mice by ~0.6°C, and this result was further confirmed by immunostaining and immunoblotting of uncoupling protein 1 (UCP1) in the BAT and the mRNA expression of thermogenic markers in BAT and subcutaneous WAT (Sub WAT) (Fig. S2C-F). However, spermidine supplementation had little effect on NC-fed lean mice in terms of weight gain, fat weight, serum glucose and insulin levels, the HOMA-IR index, and glucose tolerance (Fig. S3A-D).

### Spermidine enhanced the gut barrier function and alleviated the endotoxemia in DIO mice

**Figure 3.**
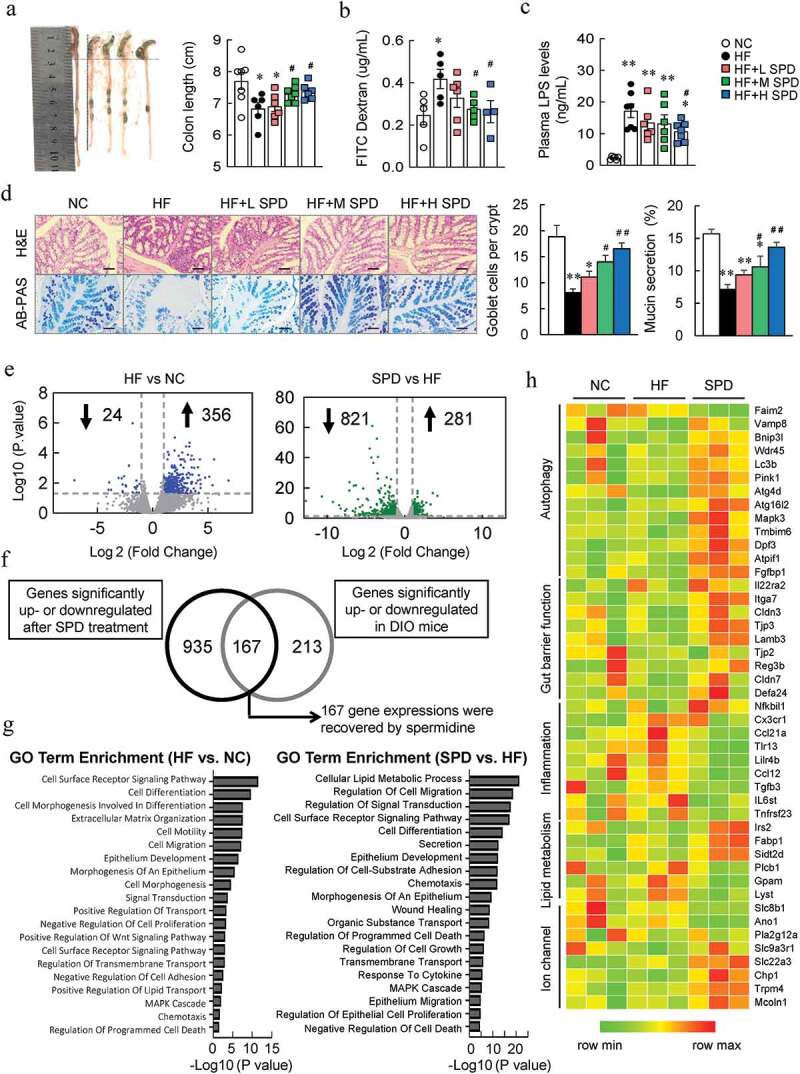
**Beneficial effects of spermidine are associated with enhanced gut barrier integrity**. (a) Representative pictures of colons and colon length (*n* = 8). (b) Intestinal permeability (*n* = 4–6). (c) Plasma LPS levels (*n* = 5–6). (d) H&E and AB-PAS staining and quantitative analysis of goblet cells and mucus secretion. Scale bar: 100 µm. (e) RNA sequencing analysis of differentially expressed genes in the colon (*n* = 4). (f) Colonic genes significantly recovered by spermidine in mice. (g) GO term enrichment analysis. (h) Unsupervised cluster analysis. Data were presented as the means ± SEM, * *p* < .05, ** *p* < .01 vs. NC; ^#^ *p* < .05, ^##^ *p* < .01 vs. HF

We next explored the effects of spermidine on barrier function. We found that the colon length was shorter in DIO mice than that in NC-fed mice, and spermidine supplementation increased the length in a dose-dependent manner (Figure 3a). FITC-labeled dextran was administrated to assess the gut permeability, and the plasma fluorescence intensities and LPS level increased significantly in DIO mice, which were decreased by spermidine (Figure 3b, c). Histological analyses of colon revealed that spermidine increased the number of mucus-secreting goblet cells and mucin secretion (Figure 3d). Interestingly, enhanced polyamine metabolism was also found in colon tissues after spermidine treatment (Fig. S1I). Similarly, spermidine did not affect barrier function in NC-fed mice (Fig. S3E and S3F).

To investigate the molecular mechanism of spermidine on barrier function, colonic transcriptomic analysis was performed. HF caused 24 genes to be significantly downregulated and 356 genes to be significantly upregulated, while spermidine treatment further caused 821 and 281 genes to be downregulated and upregulated in DIO mice, respectively (Figure 3e). Among these, a total of 167 genes was recovered by spermidine treatment (figure 3f). GO analysis indicated that pathways involved in the cell surface receptor signaling pathway, cell differentiation, epithelium development, and other related biological processes were significantly altered by an HF (Figure 3g). Notably, spermidine treatment greatly affected the pathways related to cellular lipid metabolic processes, cell migration, and signal transduction compared with those pathways in DIO mice (Figure 3g). Unsupervised cluster analysis further revealed that genes involved in autophagy and gut barrier function were increased and genes involved in inflammation were inhibited by spermidine (Figure 3h).

### Spermidine-mediated gut barrier protection was associated with autophagy induction

**Figure 4.**
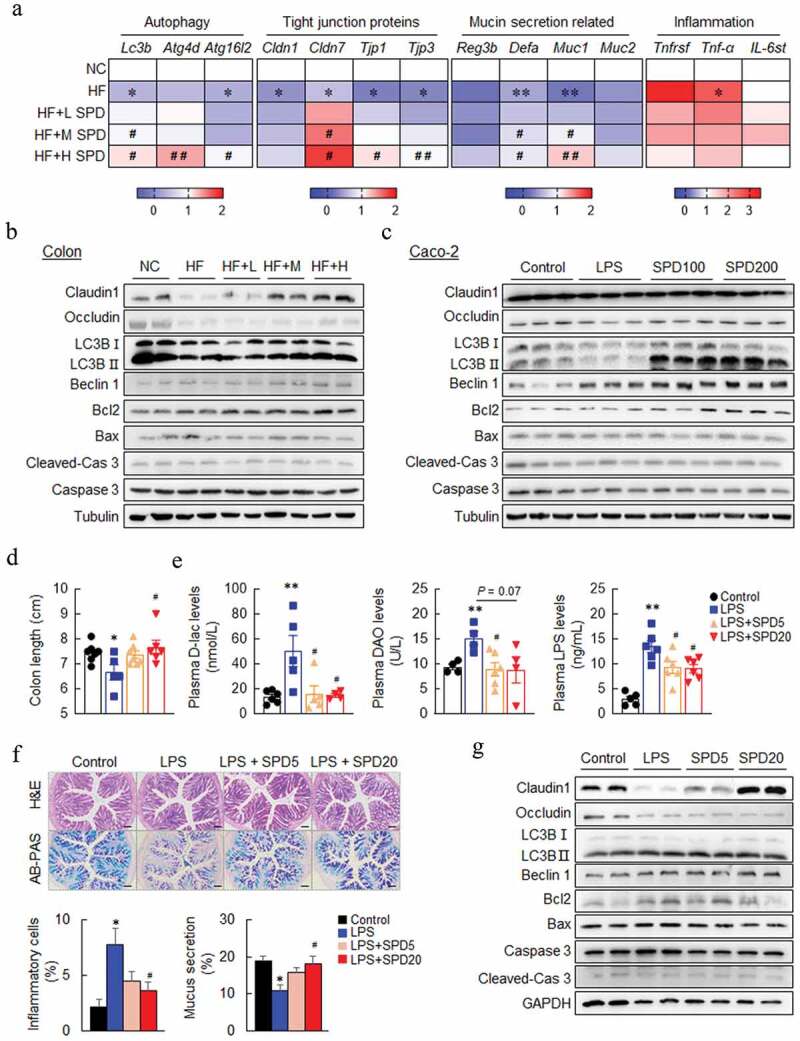
**Spermidine preserves the integrity of gut barrier associated with autophagy induction**. (a) mRNA expression of genes related to autophagy, gut barrier, mucin section and inflammation in the colon. Data were presented as the mean ± SEM, * *p* < .05, ** *p* < .01, vs. NC; ^#^ *p* < .05, ^##^ *p* < .01 < 0.01, vs. HF. (b and c) Immunoblot analysis of tight junction, autophagy and apoptosis proteins in colons of DIO mice (*n* = 5) and Caco-2 (*n* = 3). (d-f) Effect of spermidine on LPS-induced gut injury (*n* = 6). (d) Colon length of LPS-induced gut injury in mice (*n* = 6). (e) Plasma D-lactic acidosis (D-Lac), Diamine oxidase (DAO) and LPS levels (*n* = 4–6). (f) H&E and AB-PAS staining of colons and quantitative analysis of inflammatory cells infiltration and mucus secretion. Scale bar: 100 µm. (g) Immunoblot analysis of tight junction, autophagy and apoptosis proteins in colons of LPS-treated mice (*n* = 5), Data were presented as the means ± SEM, * *p* < .05, ** *p* < .01 vs. Control; ^#^ *p* < .05, ^##^ *p* < .01 vs. LPS

Next, qPCR was performed and verified that the mRNA expression of autophagy (*Lc3b, Atg4d*, and *Atg16l2*), tight junctions (*Cldn1, Cldn7, Tjp1*, and *Tjp3*), mucin secretion (*Reg3b, Defa, Muc1*, and *Muc2*), and inflammation (*Tnfrsf, Tnf-a*, and *Il6st*) markers in the colon were recovered by spermidine (Figure 4a). In addition, the protein levels of Claudin1 and Occludin were also decreased in the colon of DIO mice and were increased by spermidine (Figure 4b and Fig. S4A). Immunofluorescence staining of LC3B showed an increase in autophagy activity in the colon of spermidine-treated mice (Fig. S4B), and these results were confirmed by immunoblotting of LC3B and Beclin1 (Figure 4b and Fig. S4A). In contrast, the levels of Bcl2 were increased, and the levels of Bax and Caspase3 were decreased by spermidine (Figure 4b and Fig. S4A). Consistently, spermidine treatment increased the expression of tight junction- and autophagy-related markers and decreased the expression of apoptosis-related markers in Caco-2 cells, as assessed by qPCR and immunoblotting (Figure 4c and Fig. S5A-C). Similarly, an increase of LC3 and lysosomal associated membrane protein 1 (LAMP1) expression by spermidine were found in Caco-2 cells (Fig. S5D). However, the reduction of paracellular permeability by spermidine was not altered in the presence of the autophagy enhancer rapamycin, while it was slightly attenuated by the autophagy inhibitor 3-methyladenine (3-MA, Fig. S6A, and S6B).

Furthermore, LPS injection caused a decrease in the colon length, and it was rescued by spermidine treatment (Figure 4d). The plasma levels of D-lactate (D-Lac), diamine oxidase (DAO), and LPS were increased significantly in LPS-injected mice whereas decreased by spermidine (Figure 4e). Spermidine also decreased inflammatory cell infiltration and increased mucus secretion as assessed by histological analysis (figure 4f). The immunoblotting results also showed elevated levels of tight junction- and autophagy-related markers and down-regulated the expression of apoptosis-related markers in the colon after spermidine treatment (Figure 4g and Fig. S6C).

### Spermidine alters the composition and function of the gut microbiota

**Figure 5.**
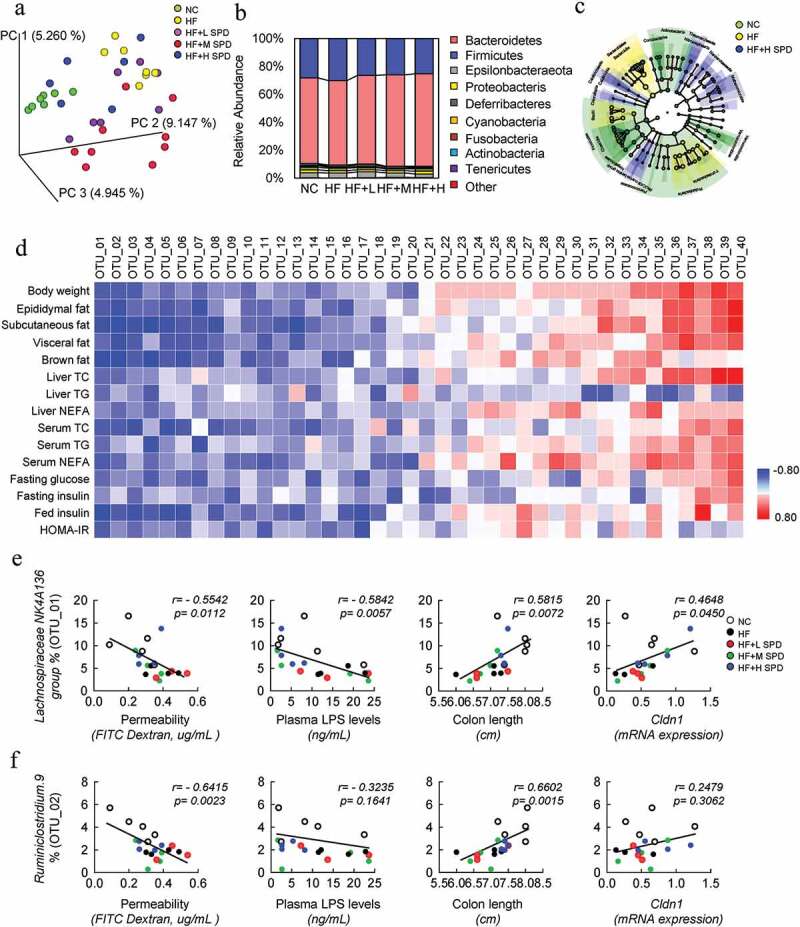
**Spermidine alters the composition and function of the gut microbiota**. (a) UniFrac principal coordinate analysis (PCoA) analysis of microbiota in cecal content (*n* = 6–8). (b) Relative abundance of microbiota at the phylum level. (c) Cladogram generated from LEfSe analysis. (d) Correlation analysis of top 40 microbes with obesity-related parameters. (e-f) Correlation analysis of *Lachnospiraceae NK4A136 group* (e) and *Ruminiclostridium.9* (f) with intestinal permeability, colon length, LPS levels and *Cldn1* expression. Values for *r* and *p* are indicated in each graph

To determine the effect of spermidine on the gut microbiota, high-throughput sequencing of 16S rRNA in the cecal content was performed. Principal coordinate analysis (PCoA) showed that the β-diversity value could be used to clearly discriminate between NC-fed lean mice and DIO mice, and spermidine treatment resulted in further discrimination compared to DIO mice, with a β-diversity value that was close to that of NC-fed mice (Figure 5a). The overall composition of gut microbiota was further analyzed at the phylum level, and the amount of *Firmicutes* increased by 4.4%, while that of *Bacteroidetes* decreased by 4.2% in DIO mice, which were rescued by spermidine (Figure 5b). LEfSe analysis revealed significant differences in the taxa found in lean, DIO- and spermidine-treated mice (Figure 5c). The functional profile of the microbial community showed a significant difference in the predicted functions between NC- and HF-fed mice as well as HF- and spermidine-treated mice (Fig. S7). These functions were mainly related to metabolism, including amino acid, nucleotide, NAD^+^ and lipid metabolism, and spermidine counteracted the alteration of these functions (Fig. S7).

Next, spearman correlation analysis was performed to identify the bacteria that might be responsible for the spermidine-mediated amelioration of metabolic phenotypes in DIO mice. We identified the top 20 bacterial genera that were negatively correlated and the top 20 genera that were positively correlated with metabolic phenotypes in DIO mice (Figure 5d and Table S1). Among these bacterial genera, SCFA-producing bacteria, including the *Lachnospiraceae NK4A136 group, Ruminiclostridium 9, Lachnospiraceae ASF356, Lachnospiraceae UCG 001*, and *Ruminiclostridium 5*, showed strong negative correlations with most of the metabolic parameters. Moreover, pathogenic bacteria including the *Rikenellaceae RC9 gut group, Alistipes, Muribaculum, Rikenella*, and *Bacteroides* displayed a strong positive association with obesity phenotypes (Figure 5d and Table S1). We further determined whether these associated bacteria were also associated with the integrity of the gut barrier and found that there was a significant negative correlation between the *Lachnospiraceae NK4A136 group* and intestinal permeability and the plasma LPS level and a positive correlation between this group and colon length and the expression of *Cldn1* (Figure 5e). *Ruminiclostridium 9* was found to be negatively correlated with intestinal permeability and positively correlated with colon length (figure 5f). However, other bacterial genera that were correlated with metabolic phenotypes were not associated with parameters related to gut barrier function (Table S2 and S3).

### Gut microbiota was required for spermidine-mediated alleviation of metabolic syndrome in DIO mice

**Figure 6.**
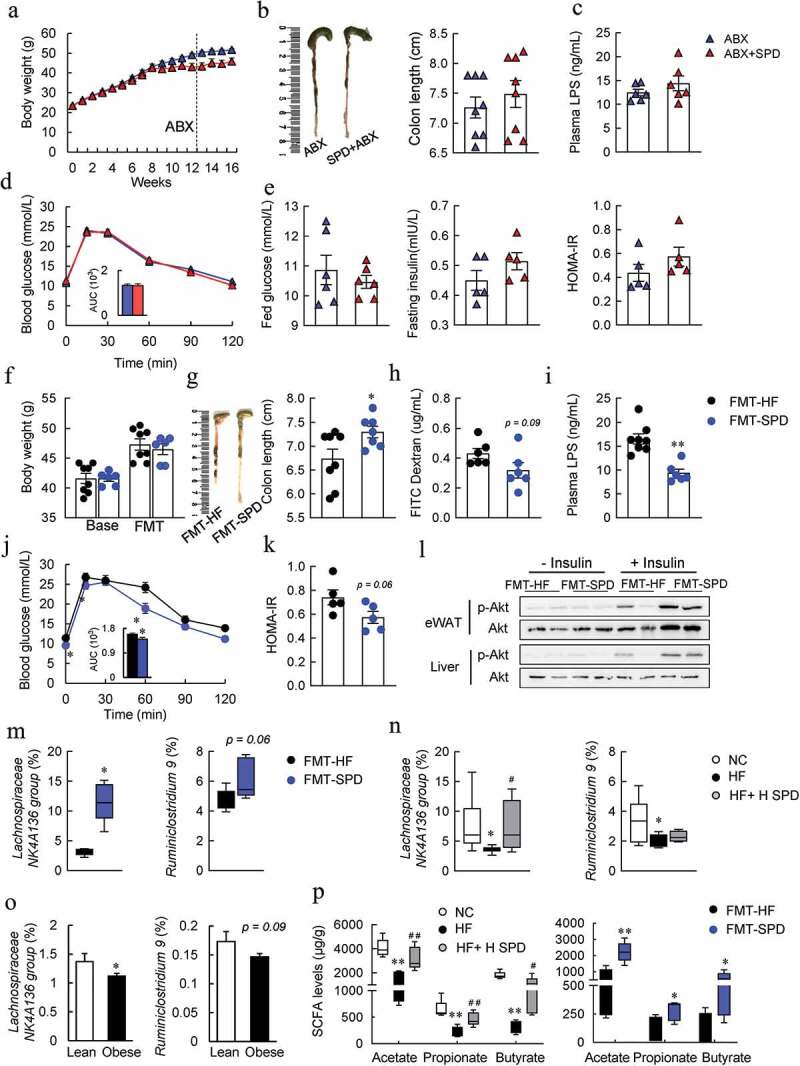
**Gut microbiota is required for spermidine-mediated alleviation of metabolic syndrome in DIO mice**. (a-e) Effects of antibiotic treatment. Mice body weight (a), colon length (b), plasma LPS levels (c), GTT (d), plasma fed glucose, fasting insulin levels and HOMA-IR index (e) (*n* = 5–6). Data were presented as the means ± SEM. (f-l) Effects of FMT treatment. Mice body weight (f), colon length (g), intestinal permeability (h), plasma LPS levels (i), GTT (j), HOMA-IR index (k) and (l) Immunoblots of phosphorylated Ser473 Akt (*p*-Akt), and Akt in the eWAT and liver of mice (*n* = 5–6). (m) Relative abundance of *Lachnospiraceae NK4A136* and *Ruminiclostridium.9* in FMT-HF and FMT-SPD groups (*n* = 5). Data were presented as the means ± SEM, * *p* < .05 vs. FMT-HF. (n) Relative abundance of *Lachnospiraceae NK4A136* and *Ruminiclostridium.9* in NC, HF and HF+H SPD groups (*n* = 6–10). * *p* < .05 vs. NC; ^#^ *p* < .05 vs. HF. (o) Relative abundance of *Lachnospiraceae NK4A136* and *Ruminiclostridium.9* in lean (*n* = 176) and obese individuals (*n* = 1005). * *p* < .05, ** *p* < .01, vs. lean. (p) Concentrations of acetate acid, propionate acid and butyrate acid in each group (*n* = 5). *p < .05 vs. NC; *^#^p* < .05 vs. HF or *p < .05 vs. FMT-HF

The microbiota and correlation analyses prompted us to further investigate whether the gut microbiota was required for spermidine-mediated alleviation of metabolic syndrome in DIO mice. Antibiotics were applied to deplete the existing microbiota in DIO- and spermidine-treated mice, which was confirmed by 16S rRNA sequencing (Fig. S8A and Fig S10A-C). Antibiotic treatment did not affect body weight in DIO- or spermidine-treated mice (Figure 6a). However, the protective effects of spermidine on colon length and endotoxemia were abolished after antibiotic treatment (Figure 6b and 6c). In addition, GTT results indicated that spermidine failed to improve glucose intolerance in antibiotic-treated mice (Figure 6d). These results were associated with the loss of the effects on reducing plasma glucose and insulin levels and the HOMA-IR index (Figure 6e), as well as BAT thermogenesis (Fig. S9A-D).

To next investigate whether the microbiota itself, especially those species whose composition and function had already been altered by spermidine, was sufficient to ameliorate the metabolic phenotypes in DIO mice, fecal microbiota transplantation (FMT) was conducted (Fig. S8B). Next, FMT was conducted, and the spermidine in feces was not affected by transplanting spermidine-altered microbiota to DIO mice (Fig. S9E), thereby ruling out the possibility that the effects exerted by FMT were attributed to the remaining spermidine in the feces of donor mice. The bodyweight of the donor mice was not changed before and after FMT of spermidine-altered microbiota (FMT-SPD) (figure 6f). FMT-SPD mice showed an increase in colon length, a slight decrease in the FITC dextran level and a significant decrease in plasma LPS levels compared to FMT of HF controlled microbiota (FMT-HF) mice (Figure 6g-i). More importantly, tight junction protein Claudin1 was enhanced whereas LC3B expression was unaffected by FMT of spermidine-altered microbiota (Fig. S9F), suggesting that the protective role for FMT may mediate through the alteration of gut microbiota, but not the activation of autophagy. Glucose intolerance, insulin resistance, and BAT thermogenesis were also slightly improved by FMT of spermidine-altered microbiota (Figure 6j-l and Fig. S9G-K).

### Increased Lachnospiraceae NK4A136 group levels might contributed to the benefits of spermidine

To determine the bacterial genera that might be responsible for the effects exerted by FMT, the composition of the gut microbiota in cecal content after FMT was further analyzed. The α-diversity, especially the Chao1, Shannon, and Margalef indexes increased significantly in FMT-SPD mice, and the β-diversity also showed clear discrimination between the FMT-treated two groups, similar with the alternations between HF and HF-SPD groups (Fig. S10A and S10B). Consistent with our previous results, the amount of *Firmicutes* decreased, whereas the amount of *Bacteroidetes* and *Deferribacteres* increased at the phylum level in FMT-SPD mice (Fig. S10C). Then, we examined the abundance of the top 40 genera that were correlated with obesity phenotypes according to previous results and found a significant increase in the *Lachnospiraceae NK4A136 group, Ruminiclostridium 5* and *Acetatifactor* and a slight increase in *Ruminiclostridium 9, Lachnospiraceae UCG-006* and *Ruminococcus 1* in FMT-SPD mice (Fig. S11A). In contrast, the abundances of the *Rikenellaceae RC9 gut group* and *Muribaculum* decreased significantly, and the abundances of *Alistipes* and *Bacteroides* tended to decrease in FMT-SPD mice (Fig. S11B). However, antibiotic treatment depleted these identified existing microbiota (Fig. S11A and S11B). Consistent with our previous data, the analyses of the correlation of the affected genera with metabolic phenotypes found that only the *Lachnospiraceae NK4A136 group* but not *Ruminiclostridium 9* was remarkably negatively correlated with permeability, plasma LPS levels and the HOMA-IR index and positively correlated with colon length (Fig. S11C and S11D). Importantly, the abundance of the *Lachnospiraceae NK4A136 group* was decreased significantly in both DIO mice and obese individuals, while spermidine treatment increased the abundance of the *Lachnospiraceae NK4A136 group* (Figure 6m-o).

On the other hand, the capability for butyrate production of the *Lachnospiraceae NK4A136 group* further prompted us to predict the microbial functions related to SCFA biosynthesis. Interestingly, these predicted functions were significantly enhanced by spermidine in both DIO mice and FMT-treated mice (Fig. S12A). Consistently, the levels of SCFAs, especially acetate, propionate, and butyrate, were significantly increased by spermidine and FMT (Figure 6p and Fig. S12B). In addition, the butyrate concentration in the feces was positively correlated with the abundance of the *Lachnospiraceae NK4A136 group* (Fig. S12 C), suggesting the pivotal role of spermidine-stimulated bacteria in the elevation of butyrate levels.

## Inhibition of TLR4 signaling contributed to anti-obesity effect spermidine

Interestingly, we found that LPS- and HF-induced the activation of TLR4 and its downstream marker Myd88 was attenuated by spermidine in both the colon and Caco-2 cells, as assessed by immunochemistry and immunoblotting (Fig. S13A-D). Functions related to LPS biosynthesis by microbiota were also attenuated in spermidine-treated and FMT mice (Fig. S13E). Molecular docking analysis further identified three binding sites for spermidine in the TLR4 protein (Fig. S13 F), indicating a link between TLR4 signaling and spermidine-mediated anti-obesity effects.

**Figure 7.**
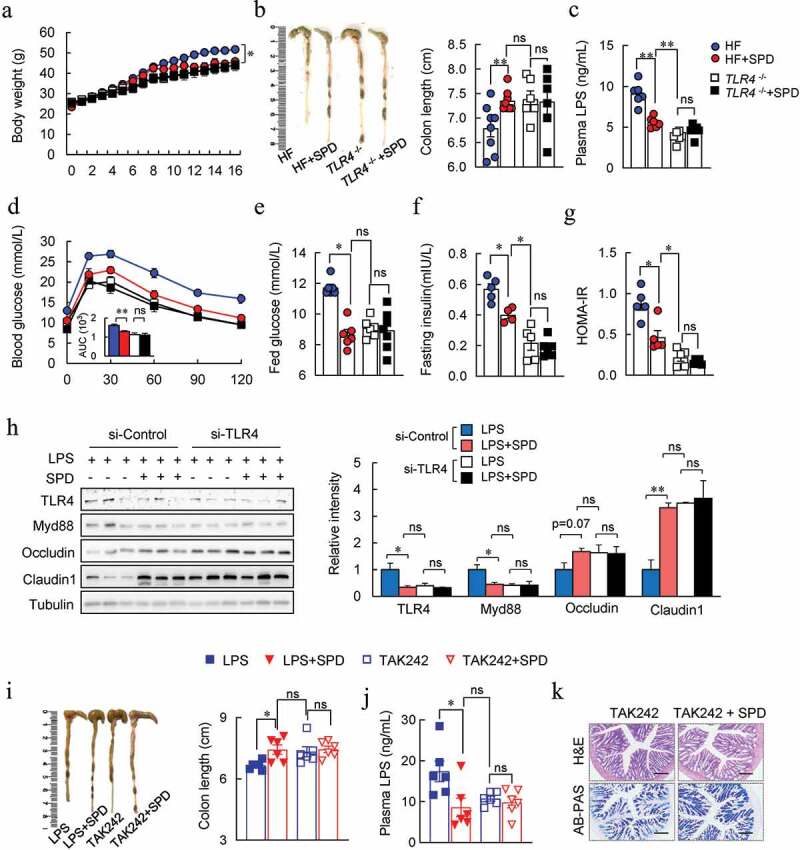
**Inhibition of TLR4 signaling contributed to anti-obesity effect spermidine**. (a-g) Effect of spermidine on *TLR4 ^−/-^* mice. Mice body weight (a), colon length (b), plasma LPS levels (c), GTT (d), fed glucose (e), fasting insulin (f), HOMA-IR (g). (h) Immunoblots of TLR4, Myd88, Occludin, Claudin1 in Caco-2 cells transfected with TLR4 siRNA. *n* = 3, experiments were repeated three times. (i) Colon length, (j) plasma LPS levels and colon H&E and AB-PAS staining (k) of TAK242 treated mice (*n* = 6). Data were presented as the means ± SEM, * *p* < .05, ** *p* < .01. ns, not significant

To verify this hypothesis, the effects of spermidine on obese TLR4^−/-^ mice were investigated. As expected, TLR4^−/-^ mice rescued HF-induced gut barrier impairment, glucose intolerance, and insulin resistance (Figure 7a-g). Spermidine-treated mice had similar effects with those in TLR4 deficient mice. However, spermidine did not affect the phenotypes of obese TLR4^−/-^ mice (Figure 7a-g). Furthermore, a similar tendency of tight junction protein expressions was found between spermidine-treated and TLR4 knockdown group in Caco-2 cells (Figure 7h). Also, TLR4 knockdown abolished spermidine-enhanced tight junction barrier (Figure 7h). Similarly, the improved effects of spermidine on LPS-induced gut barrier dysfunction in mice were abrogated after the injection of TLR4 inhibitor, TAK242 (Figure 7i-k).

## Discussion

Polyamines are involved in various biological processes and therefore have important implications for human health, especially for intestinal maturation and immune system differentiation and development.^22^ The biosynthesis of polyamines tends to decrease with age,^23,24^ and this is the reason why dietary polyamines are of importance in aged populations. Consistently, spermidine content is decreased during aging in both humans and mice, and its supplementation extends the lifespan of mice and protects against cardiovascular diseases.^25,26^ In addition, cohort study indicated that higher spermidine intake is linked to lower mortality.^27^ Although several studies have demonstrated the role of spermidine in metabolic syndrome-related diseases, including liver diseases^19,20^ and obesity,^21^ the relationship between spermidine intake and the incidence of metabolic syndrome remains unclear. Here, we found that daily spermidine intake was negatively correlated with obesity phenotypes, which serves as a theoretical basis for the establishment of recommended levels of spermidine intake for individuals with obesity.

It is believed that the high level of spermidine found in the intestinal tract originates from the diet or is synthesized by host cells and microbiota, especially spermidine in the lower parts of the intestinal tract, which is believed to be synthesized by colonic microflora.^28,29^ Microbially synthesized spermidine is partially transported to the proximal gut, while the remaining spermidine is excreted in the feces. Studies have reported that the human fecal polyamine concentration might be linked with the fecal microbiota.^28,30^ Our results also found that exogenous spermidine was mainly taken up by the intestinal tract, and HF-induced microbial alterations were accompanied by decreased levels of fecal spermidine. Importantly, the negative correlations between fecal spermidine and obesity parameters further highlight the potential therapeutic use of spermidine in the treatment of metabolic disorders.

Several studies have shown that polyamines are present at the low μM range in the blood of both humans and mice, only 7–15% of total spermidine.^28^ Thereby, it is plausible that increased spermidine intake can increase the total spermidine levels in the body, thereby causing elevated blood spermidine concentrations. For example, blood spermidine levels were significantly increased after supplementation of long term or short term of spermidine in adult mice.^25,31^ In contrast, prolonged intake of polyamine-rich products such as natto or fermented soy in humans only resulted in a slight increase of spermidine concentrations.^32^ Still, further studies that focusing on the effects of spermidine on polyamine concentration in the serum could provide more detail about the role of spermidine supplementation in maintaining human health. Since spermidine effectively alleviated fat and liver inflammation in DIO mice, it is possible that enhanced endogenous polyamines metabolism might also contribute to the amelioration of metabolic syndrome in obese mice. Furthermore, spermidine distribution data also showed that increased levels of spermidine were mainly found in the gut and feces, it is not surprising since the intestinal tissue is one of the most rapidly proliferating tissues. In this regard, it is possible that dietary spermidine might be important for the development of the intestinal tract. Thus, these support us to determine whether spermidine could protect gut barrier function and how spermidine contributes to metabolic improvement.

Mounting preclinical and clinical evidence has highlighted the critical role of the intestinal barrier and gut microbiota in metabolic disease, which has led to the revival of the “leaky gut” concept.^33^ Therefore, targeting and restoring intestinal barrier function is a tempting therapeutic approach, even though no clinical therapies currently exist. Recent advances have proposed restoring or regenerating barrier integrity by using intestinal stem cells as a therapy for repairing damaged intestinal mucosa.^34,35^ As illustrated in this study, although spermidine modestly prevented the increase of circulating LPS levels, it substantially protected the intestinal permeability as assessed by FITC-dextran. Further experiments confirmed that protection of gut barrier function by spermidine presumably due to enhanced tight junction and gut mucosal barrier, which evidenced by upregulated tight junction protein and mucin protein expressions as well as reduced DAO and D-lac levels. In addition, accumulating evidence has revealed the importance of autophagy in the physiological processes of intestinal epithelial cells and the maintenance of barrier function,^36,37^ allowing autophagy-targeting therapy to serve as another alternative for the treatment of intestinal barrier dysfunction-associated diseases. Genetic and pharmacological inhibition of the autophagy process produced an increase in body weight in obese mice, suggesting that autophagy influences the persistence of weight gain and obesity-related features.^21^ Spermidine is an autophagy inducer that protects against various declines in physiological functioning resulting from aging and cardiovascular diseases.^16,18,25^ Accordingly, our results also showed that spermidine exerted marked anti-obesity effects in DIO mice. Consistent with the role of spermidine as an autophagy inducer, we also found that spermidine treatment protected gut barrier function by activating autophagy and attenuating apoptosis in the colon and Caco-2 cells.

Excessive TLR activation stimulates signaling cascades by the host leading to systemic inflammation and insulin resistance.^6^ Particularly, TLR4 coordinates the interactions between the luminal microbiota and host metabolic genes and is thereby associated with an increased risk for metabolic syndrome.^38,39^ Studies have suggested that epithelial TLR4 activation causes the loss of barrier integrity and a leaky gut.^40,41^ The loss of epithelial TLR4 expression prevents metabolic syndrome by regulating the interactions between microbes and intestinal epithelial cells in mice.^7^ We found that spermidine treatment markedly inhibited TLR4 activation in the colon by either LPS or HF, and the protections of leaky gut and metabolic syndrome by spermidine were similar to those of TLR4 deficiency or inhibition. The presence of potential binding sites of spermidine on the TLR4 protein further supported that spermidine might act through an autophagy-independent pathway. Further studies are required to investigate the specific mechanism of how spermidine regulates TLR4 signaling, such as using conditional TLR4 knockout models.

**Figure 8.**
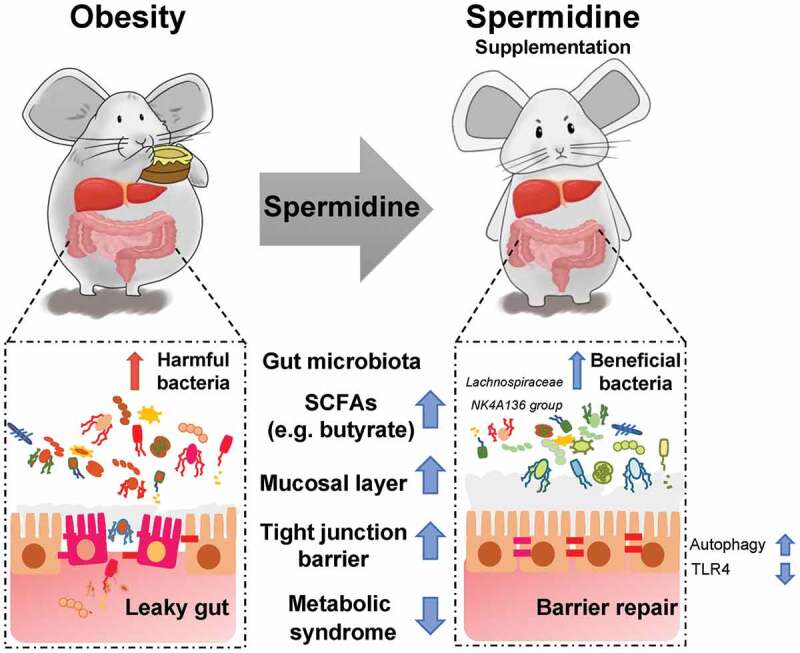
The beneficial effects of spermidine were attributable in part to the enhancement of gut barrier integrity and the alteration of the gut microbiota. Mechanistically, increased abundance of SCFA-producing bacteria *Lachnospiraceae NK4A136 group* and regulation of gut barrier function via autophagy-dependent and autophagy-independent pathways underlies the anti-obesity effects of spermidine

Numerous works have demonstrated the role and therapeutic potential of the microbiota in obesity and metabolic diseases,^42–44^ demonstrating that probiotics, prebiotics, and functional compounds are extremely important approaches for dietary intervention during the weight loss process. In the present study, we found that spermidine treatment significantly altered the composition and function of the gut microbiota in obese mice. These effects were lost after the depletion of the gut microbiota by antibiotics and restored by the transplantation of spermidine-treated microbiota into obese mice, suggesting that altered microbiota are involved in spermidine-mediated anti-obesity effects in addition to the effects of spermidine itself. In particular, the *Lachnospiraceae NK4A136 group*, representing kind of butyrate-producing bacteria, has been found to maintain gut barrier integrity in mice and is negatively correlated with intestinal permeability.^45^ Butyrate, as one of the main SCFAs produced by microbiota, is important in maintaining gastrointestinal health due to its ability to enhance epithelial barrier integrity and inhibit inflammation.^46^ In line with this finding, our data showed that the butyrate abundance was negatively correlated with obesity parameters and intestinal permeability, and an increase in the abundance of the *Lachnospiraceae NK4A136 group* caused by spermidine treatment, which was associated with increased levels of butyrate and other SCFAs. These results supported the notion that the *Lachnospiraceae NK4A136 group* contributes to the beneficial effects of spermidine. Importantly, the decreased abundance of the *Lachnospiraceae NK4A136 group* further highlights the potential benefits of approaches that target this bacterial genus. In summary, our study demonstrated that spermidine supplementation is an effective strategy for anti-obesity therapy. The beneficial effects of spermidine were attributable in part to the enhancement of gut barrier integrity by autophagy-dependent and autophagy-independent pathways and the alteration of the gut microbiota (Figure 8). Notably, our data highlighted that the improved barrier function and metabolism phenotype observed following spermidine treatment might be associated with increased *Lachnospiraceae NK4A136 group* levels. However, the clinical use and safety of spermidine for treating obesity and related metabolic syndromes still need to be investigated in the future.

## Materials and methods

### Collection and analysis of clinical and spermidine intake data

The characteristics and dietary intake data for 5799 samples were collected from the National Health and Nutrition Examination Survey (NHANES, 2009–2010).^47^ The daily intake of spermidine via the diet was estimated by referring to a polyamine database compiled by Kiechl.^27^ Spermidine intake was calculated by a special program written by our group (https://github.com/solarise94/SpermidineCalculate). The characteristics of 1908 participants without diabetes (insulin ≤17 U/L and glycosylated hemoglobin ≤7%) were used to analyze the relationship of spermidine intake with serum glucose, insulin, and HbA1c levels, and the HOMA-IR index. Data were analyzed with Python version 3.7.3, and correlation statistical analyses were performed by Pearson’s correlation by using the R statistical software (R version 3.5.3).

### Animals model

Eight-week-old male C57BL/6 J mice (China National Laboratory Animal Resource Center, Shanghai, China) were divided into four groups (*n* = 8 each group) and fed for 16 weeks as follows: normal chow (NC, P1101 F-25, Salcom Co., Ltd, Shanghai, China); high-fat diet (HF, 60% fat, Research Diets Inc., D12492, New Brunswick, NJ); HF with 5, 10, 20 mg/kg spermidine (SPD, Sigma) in drinking water (HF+ L/M/H SPD). Mice drinking water were weighted and refreshed every two days. The concentration of spermidine in drinking water has to be converted to w/v and adjust based on the changes of body weight and water consumption. TLR4^−/-^ and wild type C57BL/6 J mice (*n* = 8 each group, Model Animal Research Center of Nanjing University, Nanjing, China) were fed HF with or without 20 mg/kg SPD for 16 weeks.

For antibiotic treatment, mice were fed an HF or HF+SPD (20 mg/kg, *n* = 8 each group) for 12 weeks and then treated with ABX for 4 weeks to deplete the gut microbiota (Fig. S8A). Antibiotics (ABX) were administered in the drinking water ad libitum according to the protocol described previously.^48^ For fecal microbiota transplantation (FMT) experiment, donor mice were fed with HF or HF+SPD (20 mg/kg, *n* = 16 each group) for 12 weeks, and recipient mice (*n* = 8 each group) were fed with HF for 12 weeks and orally gavaged with antibiotic mixture for three consecutive days to deplete the microbiota. FMT was conducted as described previously,^48^ and the recipient mice were given 1 g/kg fecal suspension for 4 weeks (Supplementary Fig. 9).

For lipopolysaccharide (LPS)-induced acute gut injury (*n* = 6 each group), mice were intraperitoneal injected with 1 mg/kg/day LPS (L2630, Sigma) and gavaged with 5, 20 mg/kg SPD for 5 days. For pharmacological TLR4 inhibition (*n* = 6 each group), LPS- (1 mg/kg/day) and LPS+SPD- (20 mg/kg/day) treated mice were injected together with TLR4 inhibitor TAK242 (MCE, 2 mg/kg/day) for 5 days. All experiments were approved by the laboratory animals ethical committee of the Zhejiang University of Technology and followed NIH guide for laboratory animals (NIH Publication No. 85–23, revised 1996) for the care and use of animals.

### Spermidine measurements

Spermidine was detected by HPLC/MS according to methods described previously with slight modifications.^49^ All experiments were carried out on an Agilent 1290 Infinity Series and Triple Quadrupole 6420 from Agilent Technology (Santa Clara, CA, USA). The final data were normalized according to the tissue weight.

### 16S rRNA sequencing and data analysis

Illumina HiSeq sequencing of cecal microbiota was conducted (Novogene, Tianjin, China). The composition of the gut microbiome was determined by the Illumina HiSeq platform and QIIME2 bioinformatic analysis as previously described.^50^ The accession number for the sequenced data reported in this paper is NCBI Sequence Read Archive (SRA): PRJNA628805. For human microbiota data assessment, Hispanic/Latino individuals (ages 18–74 years at the time of recruitment during 2008–2011) were provided by a study by Kaplan.^51^ Samples from individuals identified as lean (*n* = 176) and obese (*n* = 1005) without diabetic conditions were assessed according to the Qiita study (ID 11666) (https://qiita.ucsd.edu/study/description/11666#).

### Colonic transcriptome analysis

The transcriptomic analysis of the colon was then performed by Majorbio (Shanghai, China). The gene-level read counts were generated by using STAR and NCBI gene annotation. Differential expression analysis between groups was carried out using DESeq2 and GO enrichment analysis of the differentially expressed genes was processed with DAVID (Huang da et al., 2009; Love et al., 2014).

### Cell culture

The Caco-2 cells (H010, ChuanQiu Biotechnology Co., Ltd) were stimulated with 100 ng/mL LPS alone or combined with spermidine at the indicated concentrations (50, 100, and 200 μM) for 5 days. For rapamycin or 3-methyladenine (3 MA) treatment, Caco-2 cells were treated with 100 nM rapamycin (Rapa, S1842, Beyotime) or 5 mM 3-MA (M129496, Aladdin) together with 100 ng/mL LPS and 200 μM SPD for 5 days. TLR4 was knocked down by specific siRNAs (sc-40260), which was transfected using Lipofectamine 3000 reagent following the manufacturer’s instructions. Immunofluorescence staining of Caco-2 cells was performed using antibodies against LC3B (M152-3, MBL) and LAMP1 (9091, Cell Signaling Technology).

### Metabolic analysis and histological examination

Plasma insulin and hepatic triglyceride and cholesterol were measured using commercial kits. Glucose tolerance test (GTT), insulin tolerance test (ITT) and histological analyses, including hematoxylin & eosin (H&E) and Alcian blue-periodic acid Schiff (AB-PAS) staining, immunohischemistry of TLR4 (ab13867, Abcam), F4/80 (GB11027, Servicebio), and UCP1 (ab155117, Abcam), immunofluorescence staining of LC3 (14600-1-AP, Proteintech), were performed as described previously.^52^ Adipocyte size was determined from H&E stained sections and the number of CLSs was manually counted from F4/80-stained sections in five random fields from 5 mice per group by Image J software. Intestinal permeability was assessed *in vivo* following oral administration of fluorescein-isothiocyanate (FITC)-dextran (46944–500 MG-F, Sigma). Fecal SCFAs levels were detected by GC/MS (Thermo, TRACE 1310-ISQ LT), and the final data were normalized according to the fecal weight.

### Quantitative real-time PCR

Quantitative real-time PCR (qPCR) was performed as described previously.^53^ The primer sequences are shown in Table S4.

### Immunoblotting

Immunoblots were conducted as described previously.^52^ Antibodies are shown in Table S5.

### Statistical analysis

All data are presented as means ± SEM. Differences between the mean values from two groups were assessed using two-tailed Student’s t-tests. Correlation statistical analyses are performed by Pearson’s correlation by using the R statistical software (R version 3.5.3). Differences in mean values among more than two groups were determined using ANOVA. *p* values < .05 were considered to indicate statistical significance.

## Supplementary Material

Supplemental Material

## References

[1] Bluher M. Obesity: global epidemiology and pathogenesis. Nat Rev Endocrinol. 2019;15:288–298. 30814686 10.1038/s41574-019-0176-8

[2] Ke X, Walker A, Haange SB, Lagkouvardos I, Liu Y, Schmitt-Kopplin P, von Bergen M, Jehmlich N, He X, Clavel T, et al. Synbiotic-driven improvement of metabolic disturbances is associated with changes in the gut microbiome in diet-induced obese mice. Mol Metab. 2019;22:96–109. doi:10.1016/j.molmet.2019.01.012. 30792016 PMC6437638

[3] Fang X, Wei J, He X, Lian J, Han D, An P, Zhou T, Liu S, Wang F, Min J. Quantitative association between body mass index and the risk of cancer: A global meta-analysis of prospective cohort studies. Int J Cancer. 2018;7:1595–1603. doi:10.1002/ijc.31553 29696630

[4] Thaiss CA, Levy M, Grosheva I, Zheng D, Soffer E, Blacher E, Braverman S, Tengeler AC, Barak O, Elazar M, et al. Hyperglycemia drives intestinal barrier dysfunction and risk for enteric infection. Science. 2018;359(6382):1376–1383. doi:10.1126/science.aar3318. 29519916

[5] Sun L, Ma L, Ma Y, Zhang F, Zhao C, Nie Y. Insights into the role of gut microbiota in obesity: pathogenesis, mechanisms, and therapeutic perspectives. Protein Cell. 2018;9(5):397–403. doi:10.1007/s13238-018-0546-3. 29725936 PMC5960470

[6] Cani PD, Amar J, Iglesias MA, Poggi M, Knauf C, Bastelica D, Neyrinck AM, Fava F, Tuohy KM, Chabo C, et al. Metabolic endotoxemia initiates obesity and insulin resistance. Diabetes. 2007;56(7):1761–1772. doi:10.2337/db06-1491. 17456850

[7] Lu P, Sodhi CP, Yamaguchi Y, Jia H, Prindle T Jr., Fulton WB, Vikram A, Bibby KJ, Morowitz MJ, Hackam DJ, et al. Intestinal epithelial Toll-like receptor 4 prevents metabolic syndrome by regulating interactions between microbes and intestinal epithelial cells in mice. Mucosal Immunol. 2018;11(3):727–740. doi:10.1038/mi.2017.114. 29363671 PMC6131112

[8] Luoto R, Collado MC, Salminen S, Isolauri E. Reshaping the gut microbiota at an early age: functional impact on obesity risk? Ann Nutr Metab. 2013;63(Suppl 2):17–26. doi:10.1159/000354896. 24217033

[9] Mithieux G. Gut Microbiota and Host Metabolism: what Relationship. Neuroendocrinology. 2018;106(4):352–356. doi:10.1159/000484526. 29065411

[10] Meroni M, Longo M, Dongiovanni P. The role of probiotics in nonalcoholic fatty liver disease: a new insight into therapeutic strategies. Nutrients. 2019;11(11):2642. doi:10.3390/nu11112642. PMC6893730 31689910

[11] Canfora EE, Meex RCR, Venema K, Blaak EE. Gut microbial metabolites in obesity, NAFLD and T2DM. Nat Rev Endocrinol. 2019;15(5):261–273. doi:10.1038/s41574-019-0156-z. 30670819

[12] Louis P, Flint HJ. Formation of propionate and butyrate by the human colonic microbiota. Environ Microbiol. 2017;19(1):29–41. doi:10.1111/1462-2920.13589. 27928878

[13] Zhao Y, Chen F, Wu W, Sun M, Bilotta AJ, Yao S, Xiao Y, Huang X, Eaves-Pyles TD, Golovko G, et al. GPR43 mediates microbiota metabolite SCFA regulation of antimicrobial peptide expression in intestinal epithelial cells via activation of mTOR and STAT3. Mucosal Immunol. 2018;11(3):752–762. doi:10.1038/mi.2017.118. 29411774 PMC5976519

[14] Fandriks L. Roles of the gut in the metabolic syndrome: an overview. J Intern Med. 2017;281(4):319–336. doi:10.1111/joim.12584. 27991713

[15] Stanislawski MA, Dabelea D, Lange LA, Wagner BD, Lozupone CA. Gut microbiota phenotypes of obesity. NPJ Biofilms Microbiomes. 2019;5(1):18. doi:10.1038/s41522-019-0091-8. 31285833 PMC6603011

[16] Madeo F, Eisenberg T, Pietrocola F, Kroemer G. Spermidine in health and disease. Science. 2018;359(6374):eaan2788. doi:10.1126/science.aan2788. 29371440

[17] Pietrocola F, Castoldi F, Kepp O, Carmona-Gutierrez D, Madeo F, Kroemer G. Spermidine reduces cancer-related mortality in humans. Autophagy. 2019;15(2):362–365. doi:10.1080/15548627.2018.1539592. 30354939 PMC6333461

[18] Madeo F, Bauer MA, Carmona-Gutierrez D, Kroemer G. Spermidine: a physiological autophagy inducer acting as an anti-aging vitamin in humans? Autophagy. 2019;15(1):165–168. doi:10.1080/15548627.2018.1530929. 30306826 PMC6287690

[19] Liu P, de la Vega MR, Dodson M, Yue F, Shi B, Fang D, Chapman E, Liu L, Zhang D. Spermidine Confers liver protection by enhancing NRF2 signaling through a MAP1S-mediated noncano-nical mechanism. Hepatology. 2019;70:372–388. doi:10.1002/hep.30616 PMC6597327 30873635

[20] Yue F, Li W, Zou J, Jiang X, Xu G, Huang H, Liu L. Spermidine prolongs lifespan and prevents liver fibrosis and hepatocellular carcinoma by activating MAP1S-mediated autophagy. Cancer Res. 2017;77(11):2938–2951. doi:10.1158/0008-5472.CAN-16-3462. 28386016 PMC5489339

[21] Fernandez AF, Barcena C, Martinez-Garcia GG, Tamargo-Gomez I, Suarez MF, Pietrocola F, Castoldi F, Esteban L, Sierra-Filardi E, Boya P. Autophagy counteracts weight gain, lipotoxicity and pancreatic beta-cell death upon hypercaloric pro-diabetic regimens. Cell Death Dis. 2017;8(8):e2970. doi:10.1038/cddis.2017.373. 28771229 PMC5596561

[22] Munoz-Esparza NC, Latorre-Moratalla ML, Comas-Baste O, Toro-Funes N, Veciana-Nogues MT, Vidal-Carou MC. Polyamines in food. Front Nutr. 2019;6:108. doi:10.3389/fnut.2019.00108. 31355206 PMC6637774

[23] Minois N, Carmona-Gutierrez D, Madeo F. Polyamines in aging and disease. Aging (Albany NY). 2011;3(8):716–732. doi:10.18632/aging.100361. 21869457 PMC3184975

[24] Nishimura K, Shiina R, Kashiwagi K, Igarashi K. Decrease in polyamines with aging and their ingestion from food and drink. J Biochem. 2006;139(1):81–90. doi:10.1093/jb/mvj003. 16428322

[25] Eisenberg T, Abdellatif M, Schroeder S, Primessnig U, Stekovic S, Pendl T, Harger A, Schipke J, Zimmermann A, Schmidt A, et al. Cardioprotection and lifespan extension by the natural polyamine spermidine. Nat Med. 2016;22:1428–1438. doi:10.1038/nm.4222 PMC5806691 27841876

[26] Scalabrino G, Ferioli ME. Polyamines in mammalian ageing: an oncological problem, too? A review. Mech Ageing Dev. 1984;26(2–3):149–164. doi:10.1016/0047-6374(84)90090-3. 6384679

[27] Kiechl S, Pechlaner R, Willeit P, Notdurfter M, Paulweber B, Willeit K, Werner P, Ruckenstuhl C, Iglseder B, Weger S, et al. Higher spermidine intake is linked to lower mortality: a prospective population-based study. Am J Clin Nutr. 2018;108(2):371–380. doi:10.1093/ajcn/nqy102. 29955838

[28] Ramos-Molina B, Queipo-Ortuno MI, Lambertos A, Tinahones FJ, Penafiel R. Dietary and gut microbiota polyamines in obesity- and age-related diseases. Front Nutr. 2019;6:24. doi:10.3389/fnut.2019.00024. 30923709 PMC6426781

[29] Postler TS, Ghosh S. Understanding the holobiont: how microbial metabolites affect human health and shape the immune system. Cell Metab. 2017;26(1):110–130. doi:10.1016/j.cmet.2017.05.008. 28625867 PMC5535818

[30] Matsumoto M, Benno Y. The relationship between microbiota and polyamine concentration in the human intestine: a pilot study. Microbiol Immunol. 2007;51(1):25–35. doi:10.1111/j.1348-0421.2007.tb03887.x. 17237596

[31] Schwarz C, Stekovic S, Wirth M, Benson G, Royer P, Sigrist SJ, Pieber T, Dammbrueck C, Magnes C, Eisenberg T, et al. Safety and tolerability of spermidine supplementation in mice and older adults with subjective cognitive decline. Aging (Albany NY). 2018;10(1):19–33. doi:10.18632/aging.101354. 29315079 PMC5807086

[32] Soda K, Kano Y, Sakuragi M, Takao K, Lefor A, Konishi F. Long-term oral polyamine intake increases blood polyamine concentrations. J Nutr Sci Vitaminol (Tokyo). 2009;55(4):361–366. doi:10.3177/jnsv.55.361. 19763038

[33] Chakaroun RM, Massier L, Kovacs P. Gut microbiome, intestinal permeability, and tissue bacteria in metabolic disease: perpetrators or bystanders? Nutrients. 2020;12(4):1082. doi:10.3390/nu12041082. PMC7230435 32295104

[34] Sato T, Vries RG, Snippert HJ, van de Wetering M, Barker N, Stange DE, van Es J, Abo A, Kujala P, Peters PJ, et al. Single Lgr5 stem cells build crypt-villus structures in vitro without a mesenchymalniche. Nature. 2009;459:262–265. doi:10.1038/nature07935 19329995

[35] Yui S, Nakamura T, Sato T, Nemoto Y, Mizutani T, Zheng X, Ichinose S, Nagaishi T, Okamoto R, Tsuchiya K, et al. Functional engraftment of colon epithelium expanded in vitro from a single adult Lgr5+ stem cell. Nat Med. 2012;18(4):618–623. doi:10.1038/nm.2695. 22406745

[36] Wu Y, Tang L, Wang B, Sun Q, Zhao P, Li W. The role of autophagy in maintaining intestinal mucosal barrier. J Cell Physiol. 2019;234(11):19406–19419. doi:10.1002/jcp.28722. 31020664

[37] Nighot PK, Hu CA, Ma TY. Autophagy enhances intestinal epithelial tight junction barrier function by targeting claudin-2 protein degradation. J Biol Chem. 2015;290(11):7234–7246. doi:10.1074/jbc.M114.597492. 25616664 PMC4358142

[38] Steinhardt AP, Aranguren F, Tellechea ML, Gomez Rosso LA, Brites FD, Martinez-Larrad MT, Serrano-Ríos M, Frechtel GD, Taverna MJ. A functional nonsynonymous toll-like receptor 4 gene polymorphism is associated with metabolic syndrome, surrogates of insulin resistance, and syndromes of lipid accumulation. Metabolism. 2010;59(5):711–717. doi:10.1016/j.metabol.2009.09.015. 19922963

[39] Jialal I, Huet BA, Kaur H, Chien A, Devaraj S. Increased toll-like receptor activity in patients with metabolic syndrome. Diabetes Care. 2012;35(4):900–904. doi:10.2337/dc11-2375. 22357188 PMC3308307

[40] Burgueno JF, Abreu MT. Epithelial Toll-like receptors and their role in gut homeostasis and disease. Nat Rev Gastroenterol Hepatol. 2020;17:263–278. 32103203 10.1038/s41575-019-0261-4

[41] Nighot M, Rawat M, Al-Sadi R, Castillo EF, Nighot P, Ma TY. Lipopolysaccharide-induced increase in intestinal permeability is mediated by TAK-1 activation of IKK and MLCK/MYLK gene. Am J Pathol. 2019;189(4):797–812. doi:10.1016/j.ajpath.2018.12.016. 30711488 PMC6446229

[42] Sonnenburg JL, Backhed F. Diet-microbiota interactions as moderators of human metabolism. Nature. 2016;535(7610):56–64. doi:10.1038/nature18846. 27383980 PMC5991619

[43] Ko CW, Qu J, Black DD, Tso P. Regulation of intestinal lipid metabolism: current concepts and relevance to disease. Nat Rev Gastroenterol Hepatol. 2020;17:169–183. 32015520 10.1038/s41575-019-0250-7

[44] Dabke K, Hendrick G, Devkota S. The gut microbiome and metabolic syndrome. J Clin Invest. 2019;129(10):4050–4057. doi:10.1172/JCI129194. 31573550 PMC6763239

[45] Hu S, Wang J, Xu Y, Yang H, Wang J, Xue C, Yan X, Su L. Anti-inflammation effects of fucosylated chondroitin sulphate from Acaudina molpadioides by altering gut microbiota in obese mice. Food Funct. 2019;10(3):1736–1746. doi:10.1039/C8FO02364F. 30855043

[46] Hamer HM, Jonkers D, Venema K, Vanhoutvin S, Troost FJ, Brummer RJ. Review article: the role of butyrate on colonic function. Aliment Pharmacol Ther. 2008;27:104–119. 17973645 10.1111/j.1365-2036.2007.03562.x

[47] Johnson CL, Paulose-Ram R, Ogden CL, Carroll MD, Kruszon-Moran D, Dohrmann SM, Curtin LR. National health and nutrition examination survey: analytic guidelines, 1999-2010. Vital Health Stat. 2013;2:1–24. 25090154

[48] Fabbiano S, Suarez-Zamorano N, Chevalier C, Lazarevic V, Kieser S, Rigo D, Leo S, Veyrat-Durebex C, Gaïa N, Maresca M. Functional gut microbiota remodeling contributes to the caloric restriction-induced metabolic improvements. Cell Metab. 2018;28(6):907–21 e7. doi:10.1016/j.cmet.2018.08.005. 30174308 PMC6288182

[49] Eisenberg T, Knauer H, Schauer A, Buttner S, Ruckenstuhl C, Carmona-Gutierrez D, Ring J, Schroeder S, Magnes C, Antonacci L, et al. Induction of autophagy by spermidine promotes longevity. Nat Cell Biol. 2009;11(11):1305–1314. doi:10.1038/ncb1975. 19801973

[50] Ni Y, Wang Z, Ma L, Yang L, Wu T, Fu Z. Pilose antler polypeptides ameliorate inflammation and oxidative stress and improves gut microbiota in hypoxic-ischemic injured rats. Nutr Res. 2019;64:93–108. doi:10.1016/j.nutres.2019.01.005. 30802728

[51] Kaplan RC, Wang Z, Usyk M, Sotres-Alvarez D, Daviglus ML, Schneiderman N, Talaveraet GA, Gellman M, Thyagarajan B, Moon JY, et al. Gut microbiome composition in the hispanic community health study/ study of latinos is shaped by geographic relocation, environmental factors, and obesity. Genome Biol. 2019;20:219. doi 10.1186/s13059-019-1831-z PMC6824043 31672155

[52] Ni Y, Zhuge F, Nagashimada M, Nagata N, Xu L, Yamamoto S, Fuke N, Ushida Y, Suganuma H, Kaneko S, et al. Lycopene prevents the progression of lipotoxicity-induced nonalcoholic steatohepatitis by decreasing oxidative stress in mice. Free Radic Biol Med. 2019;152:571–582. doi:10.1016/j.freeradbiomed.2019.11.036. 31790829

[53] Ni Y, Yang X, Zheng L, Wang Z, Wu L, Jiang J, Yang T, Ma L, Fu Z. Lactobacillus and bifidobacterium improves physiological function and cognitive ability in aged mice by the regulation of gut microbiota. Mol Nutr Food Res. 2019;22:e1900603. doi:10.1002/mnfr.201900603 31433910

