# Supplementary material for: Spermidine improves gut barrier integrity and gut microbiota function in diet-induced obese mice
Source: Gut Microbes. 2020 Nov 5;12(1):1832857. doi: 10.1080/19490976.2020.1832857 (PMC7668533; doi:10.1080/19490976.2020.1832857)
Supplement: Supplemental Material [file KGMI_A_1832857_SM6372.zip › Supplementary information/Supplemental figure legend final.docx]

**Supplementary Figure legends**

**Supplementary Fig. 1.** Effects of spermidine on muscle weight and insulin signaling in mice. (A) Mice food and water intake (*n* = 8). (B) Muscle weight (*n* = 8). (C) Liver weight (*n* = 8). (D) Liver triglyceride and cholesterol levels (*n* = 8). (E-F) Immunoblots of phosphorylated Ser^473^ Akt (p-Akt), and Akt in the eWAT and liver (*n* = 5). (G) Quantifications of p-p65 were normalized to total protein levels in in liver and eWAT in DIO mice. (H-I) mRNA levels of *Odc*, *Srm* and *Sms* in liver, eWAT and colon tissues of NC-fed, HF-fed and spermidine-treated mice (*n* = 8). Data were presented as the means ± SEM, **p* < 0.05, ***p* < 0.01 vs. NC; *^#^p* <0.05, *^##^p <*0.01 vs. HF.

**Supplementary Fig. 2.** Effects of spermidine on thermogenic activity in mice. (A) Brown adipose tissue (BAT) weight (*n* = 8). (B) H&E staining and immunohistochemistry for UCP1 in BAT, Scale bar: 100 µm. (C) Rectal temperature of NC, HF and HF+H SPD groups (*n* = 8). (D) Immunoblots of UCP1 in the BAT (*n* = 5). (E-F) mRNA expression of thermogenic related genes in BAT and subcutaneous fat (Sub WAT). Data were presented as the means ± SEM, **p* < 0.05, ***p* < 0.01 vs. NC; *^#^p* <0.05, *^##^p <*0.01 vs. HF.

**Supplementary Fig.** **3.** Effects of spermidine on NC-fed mice. (A) Mice body weight and food intake (*n* = 8). (B) fat tissue (*n* = 8). (C) GTT (*n* = 5). (D) glucose, insulin levels and HOMA-IR (*n* = 5). (E) colon length and (F) colon H&E and AB-PAS staining. Scale bar: 100 µm. Data were presented as the means ± SEM.

**Supplementary Fig. 4.** Spermidine induces autophagy in colons of DIO mice. (A) Quantifications of Claudin1, Occludin, LC3B, Beclin1, Bcl2/Bax and Caspase3 in colons (*n* = 5). (B) Representative images of immunofluorescent staining for LC3B in the colons (*n* = 5). Scale bar: 200 µm**.** Blue, DAPI; green, LC3B. Data were presented as the means ± SEM, **p* < 0.05, ***p* < 0.01 vs. NC; *^#^p* <0.05, *^##^p <*0.01 vs. HF.

**Supplementary Fig. 5.** Spermidine induces autophagy in Caco-2 cells. (A-B) mRNA expression of tight junction proteins and autophagy and apoptosis related genes in Caco-2 cells. (C) Quantifications of Claudin1, Occludin, LC3B, Beclin1, Bcl2/Bax and Caspase3 in Caco-2 cells. *n* = 6, experiments were repeated three times. (D) Representative confocal images of LC3B (red), LAMP1(green), and DAPI (blue) in Caco-2 cell, Scale bar: 50 µm. Data were presented as the means ± SEM, **p* < 0.05, ***p* < 0.01 vs. Control; *^#^p* <0.05, *^##^p <*0.01 vs. LPS.

**Supplementary Fig. 6.** Spermidine mediated enhanced tight junction protein was associated with autophagy induction. (A) Immunoblot analysis of tight junction, autophagy and apoptosis proteins in Caco-2 treated with rapamycin. (B) Immunoblot analysis of tight junction, autophagy and apoptosis proteins in Caco-2 treated with 3-MA. *n* = 3, experiments were repeated three times. (C) Quantifications of Claudin1, Occludin, LC3B, Beclin1, Bcl2/Bax and Caspase3 in colons of LPS-induced mice. Data were presented as the means ± SEM, **p* < 0.05, ***p* < 0.01 vs. Control; *^#^p* <0.05, *^##^p <*0.01 vs. LPS; ^&^*P* < 0.05, vs. LPS+SPD.

**Supplementary Fig. 7.** Effects of spermidine on the functional profiling of microbial communities. Microbial functional profiling was predicted by PICRUSt, and significant changed functional profiles were compared between NC and HF-fed mic or HF and HF+H SPD mice.

**Supplementary Fig. 8.** Diagram of ABX treatment and FMT experimental design. (A) Mice treated with or without spermidine (20 mg/kg) under HFD for 12 weeks and were then treated with antibiotic cocktail (ABX) for 4 weeks to induce microbiota depletion (*n* = 8). (B) Donor feces samples were collected daily, followed by a series of steps and then transferred to HFD-fed mice by oral gavage for 4 weeks (*n* = 8).

**Supplementary Fig. 9.** Effects of ABX and FMT treatment on BAT thermogenic activity. (A-D) Effect of ABX on BAT thermogenic activity (*n* = 8). BAT weight (A), rectal temperature (B), H&E staining and immunohistochemistry for UCP1(C) and mRNA levels of *Ucp1* (D) in the BAT, Scale bar: 100 µm. Data were presented as the means ± SEM. (E) Spermidine concentration in the feces of FMT-HF and FMT-SPD groups (*n* = 4). (F) Immunoblots of Claudin1 and LC3B in colon of FMT-treated mice and quantification data. (G) Quantifications of p-AKT were normalized to total protein levels in eWAT and liver. (H-K) Effects of FMT on BAT thermogenic activity (*n* = 8). BAT weight (H), rectal temperature (I), H&E staining and immunohistochemistry for UCP1(J) and mRNA levels of *Ucp1* (K) in BAT, Scale bar: 100 µm. Data were presented as the means ± SEM. **p* < 0.05, ***p* < 0.001, FMT-SPD vs. FMT-HF.

**Supplementary Fig. 10.** Effects of ABX and FMT treatment on gut microbiota composition. (A) α-Diversity (n = 5). (B) UniFrac principal coordinate analysis (PCoA) (n =5). (C) Gut microbiota composition at the phylum level in cecal contents analyzed by 16S rRNA gene sequencing (n = 5). Data were presented as the means ± SEM. *p < 0.05, **p < 0.01.

**Supplementary Fig. 11.** Effects of ABX and FMT treatment on identified bacterial species. (A and B) Relative abundance of identified gut microbiota of ABX-treated and FMT-treated groups at the genus level (*n* = 5). (C and D) Correlation analysis between intestinal permeability, LPS levels, colon length and HOMA-IR with *Lachnospiraceae* *NK4A136 group* and *Ruminiclostridium 9*. Values for *r* and *p* are indicated in each graph. Data were presented as the means ± SEM, **p* < 0.05, ***p* < 0.01, FMT-SPD vs. FMT-HF.

**Supplementary Fig. 12.** (A) Gut microbiota function associated with SCFA-producing related were compared between HF and HF+SPD mic or FMT-HF and FMT-SPD mice (*n* = 5). (B) Concentrations of isobutyric acid, isovaleric acid, valeric acid and caproic acid in each group (*n* = 5). (C) Correlation analysis between the abundance of *Lachnospiraceae* *NK4A136 group* and butyrate levels. Values for *r* and *p* are indicated in graph. Data were presented as the means ± SEM, **p* < 0.05 vs. NC; *^#^p* <0.05 vs. HF.

**Supplementary Fig. 13.** Effect of spermidine on TLR4 signaling activation. (A) Immunohistochemistry for TLR4 in colon of LPS-treated mice. (B) Immunoblots of TLR4 and Myd88 in colon of LPS-treated mice. (C) Immunoblots of TLR4 and Myd88 in Caco-2 cells. (D) Immunoblots of TLR4 and Myd88 in colon of DIO mice. (E) Gut microbiota function of lipopolysaccharide biosynthesis in HF, HF+SPD, FMT-HF and FMT-SPD mice. (F) Molecular docking between spermidine with TLR4. N-Acetylglucosamine (NAG), β-D-mannopyranoside (BMA), β-L-fucose (FUL) were known as ligands for TLR4, and the binding energies were -4.4, -4.2 and -3.7 kcal/mol, respectively.
