# Supplementary material for: Spermidine improves gut barrier integrity and gut microbiota function in diet-induced obese mice
Source: Gut Microbes. 2020 Nov 5;12(1):1832857. doi: 10.1080/19490976.2020.1832857 (PMC7668533; doi:10.1080/19490976.2020.1832857)
Supplement: Supplemental Material [file KGMI_A_1832857_SM6372.zip › Supplementary information/Supplemental table final.docx]

**Supplementary Tables**

**Supplementary Table 1.** Gut microbiota identified in Figure 5D.

| **OTU** | **Microbes** | **OTU** | **Microbes** |
| --- | --- | --- | --- |
| OUT_01 | *Lachnospiraceae NK4A136 group* | OUT_21 | *Erysipelatoclostridium* |
| OUT_02 | *Ruminiclostridium 9* | OUT_22 | *Epulopiscium* |
| OUT_03 | *Lachnospiraceae ASF356* | OUT_23 | *Fusobacterium* |
| OUT_04 | *Lachnospiraceae UCG 001* | OUT_24 | *Dorea* |
| OUT_05 | *Ruminiclostridium 5* | OUT_25 | *Ruminococcaceae UCG 003* |
| OUT_06 | *LachnospiraceaeUCG 006* | OUT_26 | *Streptococcus* |
| OUT_07 | *Ruminococcu .1* | OUT_27 | *Desulfovibrio* |
| OUT_08 | *Oscillibacter* | OUT_28 | *Erysipelotrichaceae UCG 003* |
| OUT_09 | *Acetatifactor* | OUT_29 | *Holdemanella* |
| OUT_10 | *Ruminiclostridium* | OUT_30 | *Paraburkholderia* |
| OUT_11 | *Lactococcus* | OUT_31 | *Bacillus* |
| OUT_12 | *Peptococcus* | OUT_32 | *Parabacteroides* |
| OUT_13 | *Odoribacter* | OUT_33 | *Parasutterella* |
| OUT_14 | *UBA1819* | OUT_34 | *Tyzzerella* |
| OUT_15 | *Lachnospiraceae FCS020 group* | OUT_35 | *Stenotrophomonas* |
| OUT_16 | *GCA 900066575* | OUT_36 | *Bacteroides* |
| OUT_17 | *Clostridium sensu stricto 13* | OUT_37 | *Rikenella* |
| OUT_18 | *Butyricicoccus* | OUT_38 | *Muribaculum* |
| OUT_19 | *Blautia* | OUT_39 | *Alistipes* |
| OUT_20 | *Prevotellaceae UCG 001* | OUT_40 | *Rikenellaceae RC9 gut group* |

**Supplementary Table 2.** Correlation analysis of identified bacteria (OTU03-OTU10) with intestinal permeability, LPS levels, colon length and expression of *Cldn1*.

| **Microbes** | **Permeability** | **LPS levels** | **Colon length** | ***Cldn1* expression** |
| --- | --- | --- | --- | --- |
| *Lachnospiraceae ASF356* | *r= - 0.4298* | *r= - 0.3595* | *r= - 0.4966* | *r= - 0.5082* |
|  | *p= 0.0586* | *p= 0.1197* | *p= 0.0259* | *p= 0.0263* |
| *Lachnospiraceae UCG 001* | *r= - 0.3203* | *r= - 0.4080* | *r= - 0.2268* | *r= - 0.3961* |
|  | *p= 0.1686* | *p= 0.0741* | *p= 0.3362* | *p= 0.0931* |
| *Ruminiclostridium 5* | *r= - 0.2695* | *r= - 0.1608* | *r= - 0.4267* | *r= - 0.1192* |
|  | *p= 0.2507* | *p= 0.4775* | *p= 0.0606* | *p= 0.6269* |
| *Lachnospiraceae UCG 006* | *r= - 0.1625* | *r= - 0.1176* | *r= - 0.2140* | *r= - 0.2797* |
|  | *p= 0.4937* | *p= 0.6216* | *p= 0.3649* | *p= 0.2464* |
| *Ruminococcu 1* | *r= - 0.0840* | *r= - 0.0251* | *r= - 0.1338* | *r= - 0.1063* |
|  | *p= 0.7274* | *p= 0.9163* | *p= 0.5737* | *p= 0.6650* |
| *Oscillibacter* | *r= - 0.2480* | *r= - 0.1570* | *r= - 0.4192* | *r= - 0.5030* |
|  | *p= 0.2918* | *p= 0.5086* | *p= 0.0658* | *p= 0.081* |
| *Acetatifactor* | *r= - 0.2694* | *r= - 0.4443* | *r= - 0.2475* | *r= - 0.3280* |
|  | *p= 0.2507* | *p= 0.0497* | *p= 0.2927* | *p= 0.1703* |
| *Ruminiclostridium* | *r= - 0.2912* | *r= - 0.2894* | *r= - 0.3669* | *r= - 0.3398* |
|  | *p= 0.2130* | *p= 0.2195* | *p= 0.1116* | *p= 0.1546* |

**Supplementary Table 3.** Correlation analysis of identified bacteria (OTU31-OTU40) with intestinal permeability, LPS levels, colon length and expression of *Cldn1*.

| **Microbes** | **Permeability** | **LPS levels** | **Colon length** | ***Cldn1* expression** |
| --- | --- | --- | --- | --- |
| *Rikenellaceae*  *RC9 gut group* | *r= 0.0032* | *r= 0.0272* | *r= -0.1970* | *r= -0.02287* |
|  | *p= 0.9891* | *p= 0.9091* | *p= 0.4050* | *p= 0.9259* |
| *Alistipes* | *r= 0.07304* | *r= -0.2390* | *r= 0.0838* | *r= - 0.0004* |
|  | *p= 0.7596* | *p= 0.3103* | *p= 0.7254* | *p= 0.9984* |
| *Muribaculum* | *r= 0.1770* | *r= -0.02531* | *r= 0.0464* | *r= 0.1458* |
|  | *p= 0.4554* | *p= 0.9157* | *p= 0.8459* | *p= 0.5514* |
| *Rikenella* | *r= - 0.2898* | *r= 0.1528* | *r= - 0.2111* | *r= 0.1042* |
|  | *p= 0.2151* | *p= 0.5201* | *p= 0.3717* | *p= 0.6713* |
| *Bacteroides* | *r= 0.4260* | *r= 0.4077* | *r= - 0.4129* | *r= - 0.5457* |
|  | *p= 0.0611* | *p= 0.0744* | *p= 0.0704* | *p= 0.0157* |
| *Stenotrophomonas* | *r= 0.2396* | *r= 0.3663* | *r= 0.06397* | *r= 0.1519* |
|  | *p= 0.2918* | *p= 0.1122* | *p= 0.7887* | *p= 0.5347* |
| *Tyzzerella* | *r= - 0.0724* | *r= - 0.2400* | *r= - 0.0779* | *r= - 0.3031* |
|  | *p= 0.7615* | *p= 0.3082* | *p= 0.7441* | *p= 0.2072* |
| *Parasutterella* | *r= -0. 2610* | *r= -0. 5251* | *r= -0. 2610* | *r= - 0.6112* |
|  | *p= 0.2664* | *p= 0.0174* | *p= 0.2664* | *p= 0.0054* |
| *Parabacteroides* | *r= - 0.0598* | *r= -0.0181* | *r= - 0.2707* | *r= -0.2168* |
|  | *p= 0.8020* | *p= 0.9397* | *p= 0.2484* | *p= 0.3727* |
| *Bacillus* | *r= - 0.0685* | *r= 0.2513* | *r= - 0.3162* | *r=-0.2665* |
|  | *p= 0.7739* | *p= 0.2853* | *p= 0.1744* | *p= 0.2701* |

**Supplementary Table 4.** Primers sequences.

| Name | Forward Primer | Reverse Primer |
| --- | --- | --- |
| *M*. *GAPDH* | 5’-AGGTCGGTGTGAACGGATTTG-3’ | 5’-TGTAGACCATGTAGTTGAGGTCA-3’ |
| *M. Cldn1* | 5’-CTGGAAGATGATGAGGTGCAGAAGA-3’ | 5’-CCACTAATGTCGCCAGACCTGAA-3’ |
| *M. Cldn7* | 5’-GGCCTGATAGCGAGCACTG-3’ | 5’-GTGACGCACTCCATCCAGA-3’ |
| *M. LC3B* | 5’-TTATAGAGCGATACAAGGGGGAG-3’ | 5’-CGCCGTCTGATTATCTTGATGAG-3’ |
| *M. Atg4d* | 5’-GTCAAGTATGGTTGGGCAGTT-3’ | 5’-TGTCACCCTCTCCCTCGAAAT-3’ |
| *M. Atg16l2* | 5’-GGAGAGACTCAGTCCAAGGAA-3’ | 5’-CCACGTCATTGCAGTAGGAAAG-3’ |
| *M. LC3B* | 5’-TTATAGAGCGATACAAGGGGGAG-3’ | 5’-CGCCGTCTGATTATCTTGATGAG-3’ |
| *M. Tjp1* | 5’-CCACCTCTGTCCAGCTCTTC-3’ | 5’-CACCGGAGTGATGGTTTTCT-3’ |
| *M. Tjp3* | 5’-TCGGCATAGCTGTCTCTGGA-3’ | 5’-GTTGGCTGTTTTGGTGCAGG-3’ |
| *M. Rge3b* | 5’-ACTCCCTGAAGAATATACCCTCC-3’ | 5’-CGCTATTGAGCACAGATACGAG-3’ |
| *M. Defa* | 5’-CACCTTCTCATCCAGTGGCATC-3’ | 5’-TGGAGGTACAAGCACAGTGAG-3’ |
| *M. Muc1* | 5’-AGTTACGGTCAGGCTGCTCCGTGGT-3 | 5’-ACCCTCCCGGAAAACCACAGTC-3 |
| *M. Muc2* | 5’-GATGGCACCTACCTCGTTGT-3 | 5’-GTCCTGGCACTTGTTGGAAT-3 |
| *M. Tnfrsf* | 5’-CTTCAACTGTCCCGATGGTGA-3’ | 5’-AGGCCATTATCTTTCCCTGTGA-3’ |
| *M. Tnf-α* | 5’-ACACCGAGATTTCCTTCAAACTG-3’ | 5’-CCATCTAGGGTTATGATGCTCTTCA-3’ |
| *M. IL-6st* | 5’-CCGTGTGGTTACATCTACCCT-3’ | 5’-CGTGGTTCTGTTGATGACAGTG-3’ |
| *M. Ucp1* | 5’-ACTGCCACACCTCCAGTCATT-3’ | 5’-CTTTGCCTCACTCAGGATTGG-3’ |
| *M. Prdm16* | 5’-GGCGAGGAAGCTAGCCAAA-3’ | 5’-GGCGAGGAAGCTAGCCAAA-3’ |
| *M. Fabp4* | 5’-ACACCGAGATTTCCTTCAAACTG-3’ | 5’-CCATCTAGGGTTATGATGCTCTTCA-3’ |
| *M. CideA* | 5’-ATCACAACTGGCCTGGTTACG-3’ | 5’-TACTACCCGGTGTCCATTTCT-3’ |
| *M. Myod* | 5’-CGCCACTCCGGGACATAG-3’ | 5’-GAAGTCGTCTGCTGTCTCAAAGG-3’ |
| *M. Mgf5* | 5’-CAGCCCCACCTCCAACTG-3’ | 5’-GGGACCAGACAGGGCTGTTA-3’ |
| *M. Cebp6* | 5’-TTATAAACCTCCCGCTCGGC-3’ | 5’-CTCAGCTTGTCCACCGTCTT-3’ |
| *M. sms* | 5'-CACAGCACGCTCGACTTCAA-3' | 5'-TGCCATTCTTGTTCGTGTAAGTT-3' |
| *M srm* | 5'-ACATCCTCGTCTTCCGCAGTA-3' | 5'-GGCAGGTTGGCGATCATCT-3' |
| *M Odc* | 5'-GACGAGTTTGACTGCCACATC-3' | 5'-CGCAACATAGAACGCATCCTT-3' |
| *H. Actin* | 5’-GTACCACTGGCATCGTGATGGACT-3 | 5’-CCGCTCATTGCCAATGGTGAT-3 |
| *H. Atg5* | 5’-AAAGATGTGCTTCGAGATGTGT-3 | 5’-CACTTTGTCAGTTACCAACGTCA-3 |
| *H. LC3B* | 5’-GATGTCCGACTTATTCGAGAGC-3 | 5’-TTGAGCTGTAAGCGCCTTCTA-3 |
| *H. Beclin1* | 5’-CGGGAAGTCGCTGAAGACAG-3 | 5’-CCATCCTGGCGAGGAGTTTC-3 |
| *H. Bcl2* | 5’-AAGAGCAGACGGATGGAAAAAGG | 5’-GGGCAAAGAAATGCAAGTGAATG-3 |
| *H. Caspase3* | 5’-TGGCATTGAGACAGACA-3 | 5’-GGCACAAAGCGACTG-3 |
| *H. Cldn1* | 5’-AACGCGGGGCTGCAGCTGTTG-3 | 5’-GGATAGGGCCTTGGTGTTGGGT-3 |
| *H. Cldn3* | 5’-TGCTGTTCCTTCTCGCCGCC-3 | 5’-CTTAGACGTAGTCCTTGCGG-3 |
| *H. Cldn4* | 5’-GTCTGCCTGCATCTCCTCTGT-3 | 5’-CCTCTAAACCCGTCCATCCA-3 |
| *H. Occludin* | 5’-TCAGGGAATATCCACCTATCACTTCAG-3 | 5’-CATCAGCAGCAGCCATGTACTCTTCAC-3 |
| *H. Tjp1* | 5’-CGGTCCTCTGAGCCTGTAAG-3 | 5’-GGATCTACATGCGACGACAA-3 |

**Supplementary Table 5.** Antibodies for immunoblotting.

| Protein | Supplier | Catalog number | Dilution |
| --- | --- | --- | --- |
| Phospho-NF-κB p65 | Cell signaling | 3033 | 1:1000 |
| NF-κB p65 | Cell signaling | 8242 | 1:1000 |
| Phospho-Akt (Ser473) | Cell signaling | 4060 | 1:1000 |
| Akt | Cell signaling | 9272 | 1:1000 |
| Beclin-1 | Cell signaling | 3495 | 1:1000 |
| Bax | Cell signaling | 2772 | 1:1000 |
| Bcl2 | Cell signaling | 3498 | 1:1000 |
| Caspase3 | Cell signaling | 14220 | 1:1000 |
| Myd88 | Cell signaling | 4283 | 1:1000 |
| LAMP1 | Cell signaling | 9091 | 1:200 |
| Claudin1 | Abcam | ab15098 | 1:200 |
| Occludin | Abcam | ab167161 | 1:1000 |
| TLR4 | Abcam | ab13867 | 1:1000 |
| UCP1 | Abcam | ab155117 | 1:1000 |
| LC3B | MBL | m152-3 | 1:1000 |
| F4/80 | Servicebio | GB11027 | 1:200 |
| a-Tubulin | Santa Cruz Bio | sc-8035 | 1:1000 |
| β-Actin | HuaAn Bio | EM21002 | 1:1000 |
| GAPDH | HuaAn Bio | EM1101 | 1:1000 |
