# Supplementary figures and images for: Spermidine improves gut barrier integrity and gut microbiota function in diet-induced obese mice
Source: Gut Microbes. 2020 Nov 5;12(1):1832857. doi: 10.1080/19490976.2020.1832857 (PMC7668533; doi:10.1080/19490976.2020.1832857)

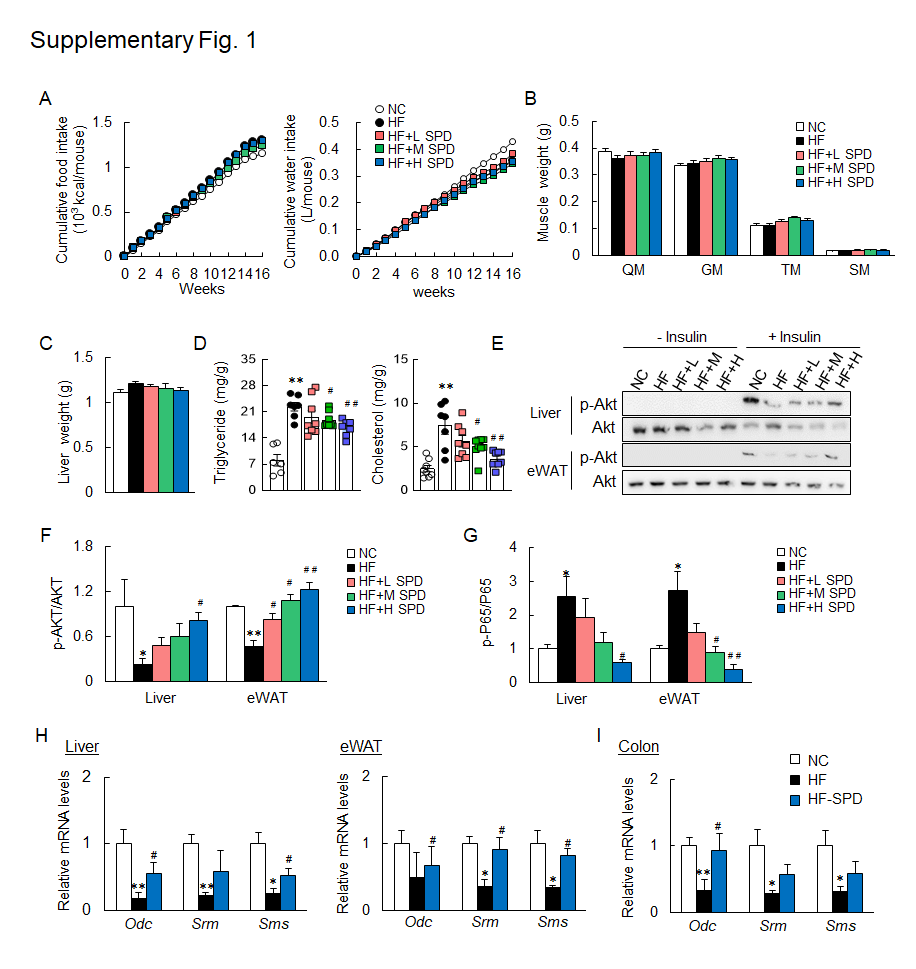

Supplement: Supplemental Material [file KGMI_A_1832857_SM6372.zip › Supplementary information/FigS1.tif]

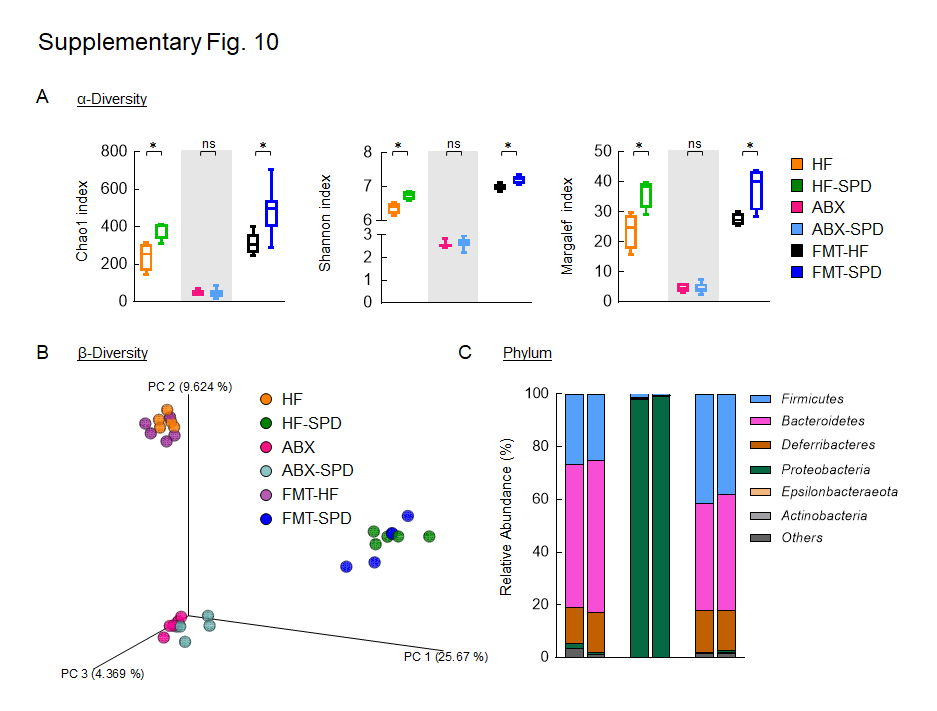

Supplement: Supplemental Material [file KGMI_A_1832857_SM6372.zip › Supplementary information/FigS10.tif]

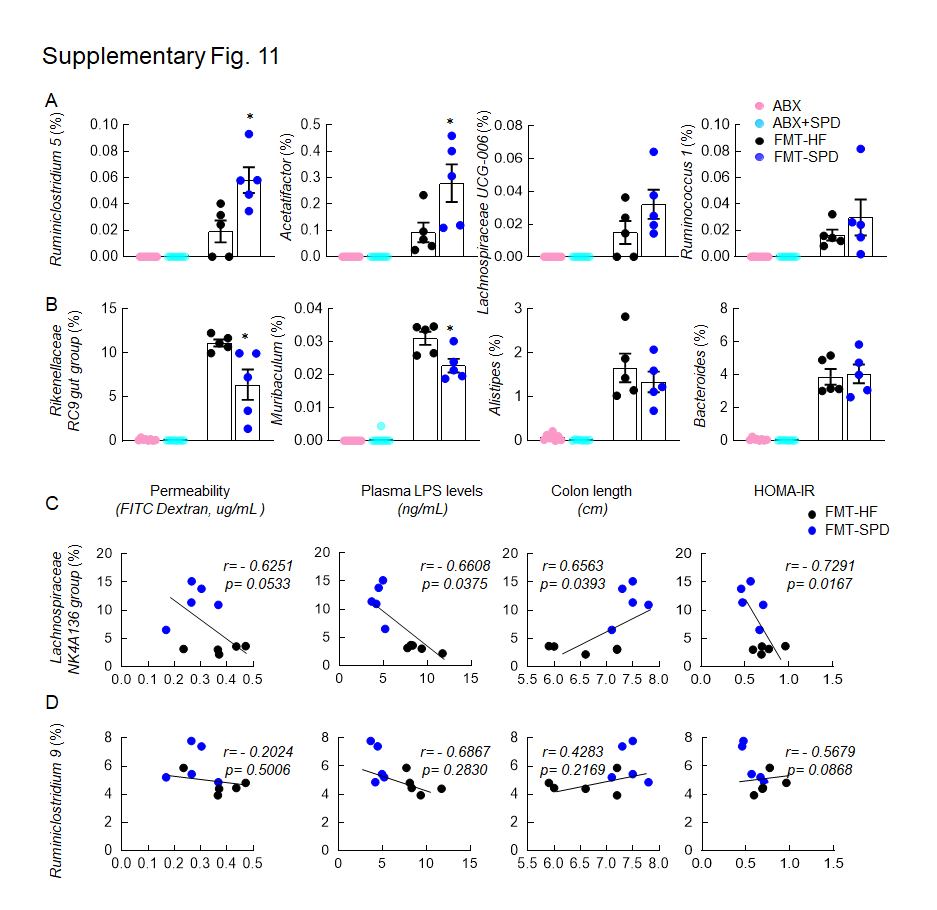

Supplement: Supplemental Material [file KGMI_A_1832857_SM6372.zip › Supplementary information/FigS11.tif]

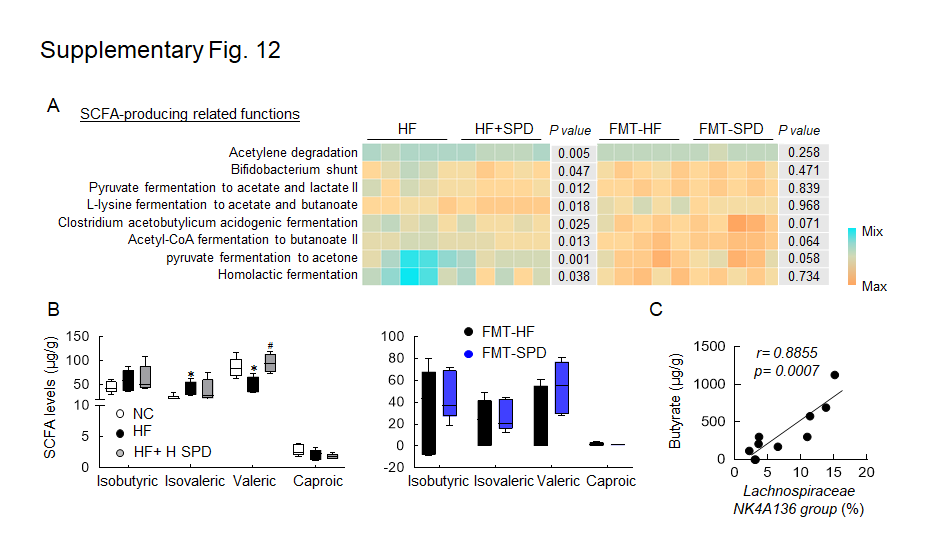

Supplement: Supplemental Material [file KGMI_A_1832857_SM6372.zip › Supplementary information/FigS12.tif]

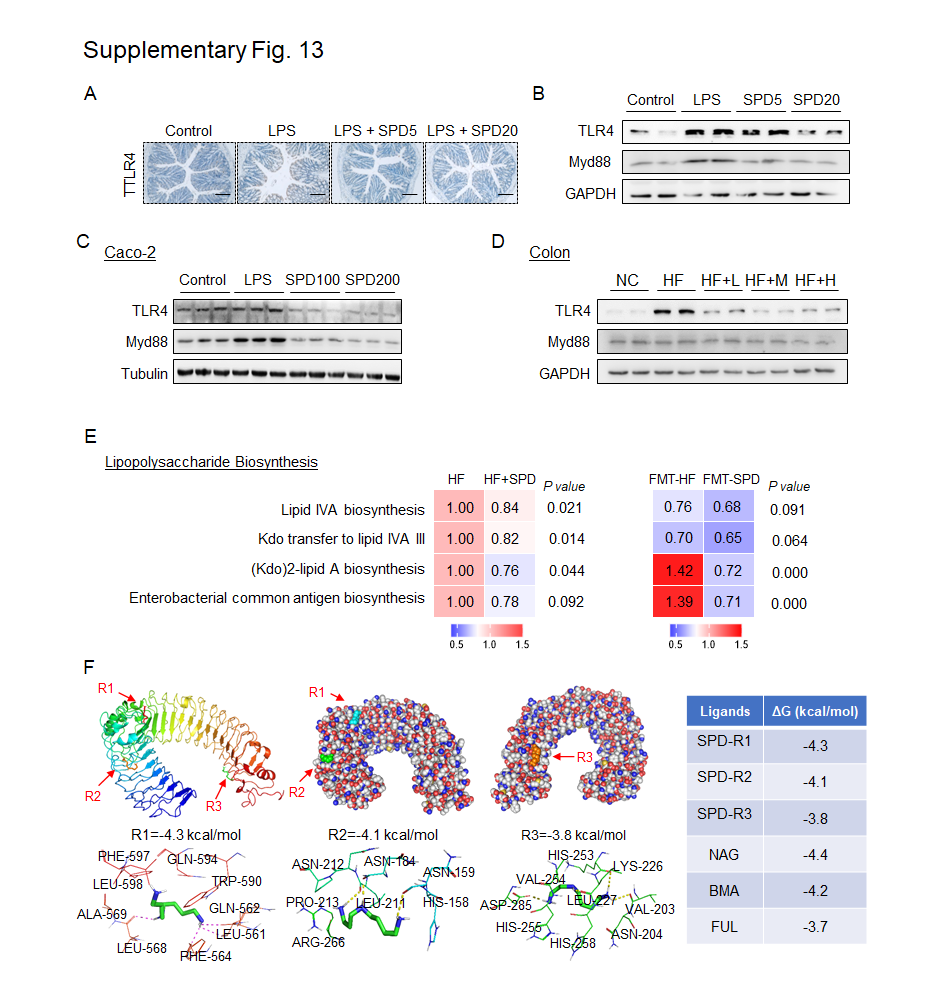

Supplement: Supplemental Material [file KGMI_A_1832857_SM6372.zip › Supplementary information/FigS13.tif]

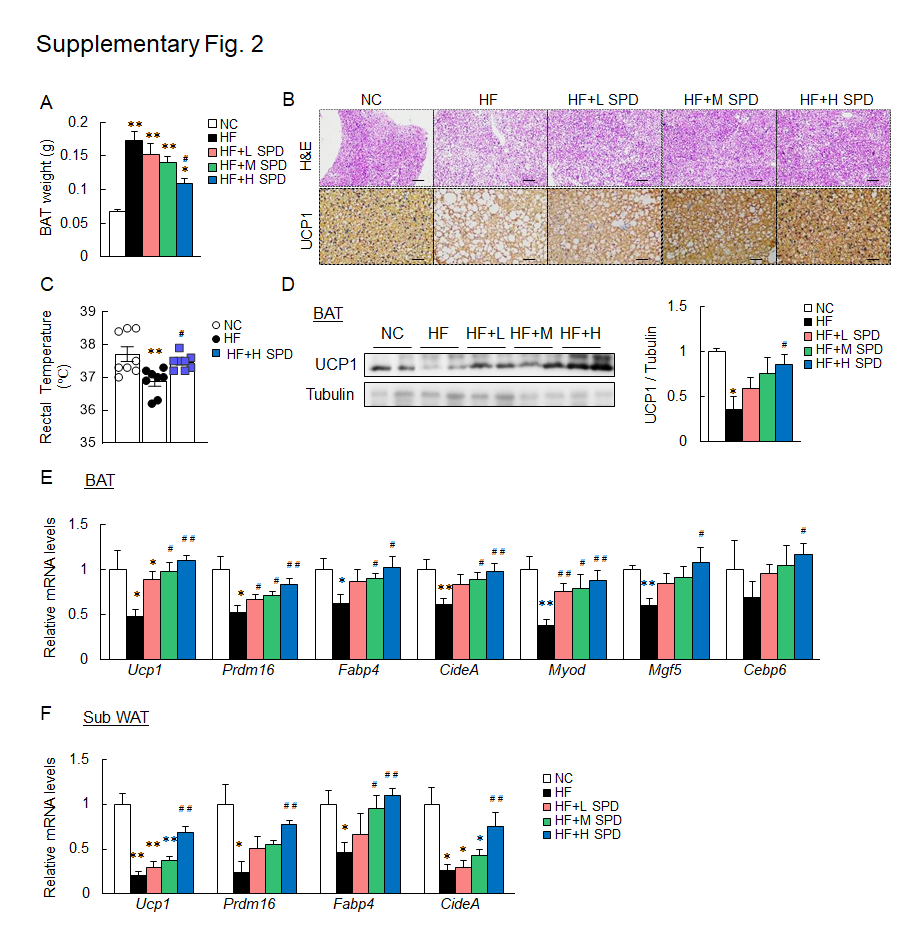

Supplement: Supplemental Material [file KGMI_A_1832857_SM6372.zip › Supplementary information/FigS2.tif]

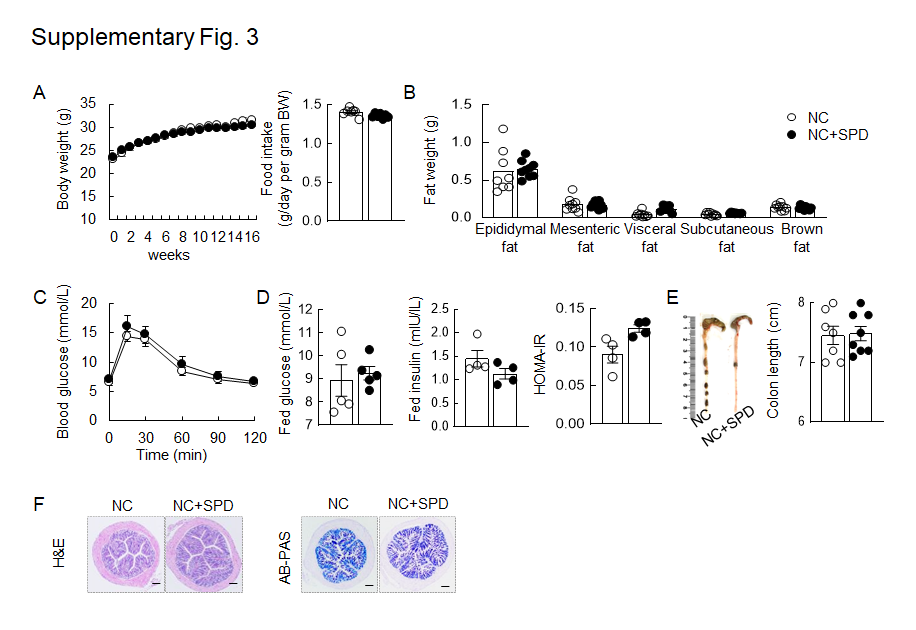

Supplement: Supplemental Material [file KGMI_A_1832857_SM6372.zip › Supplementary information/FigS3.tif]

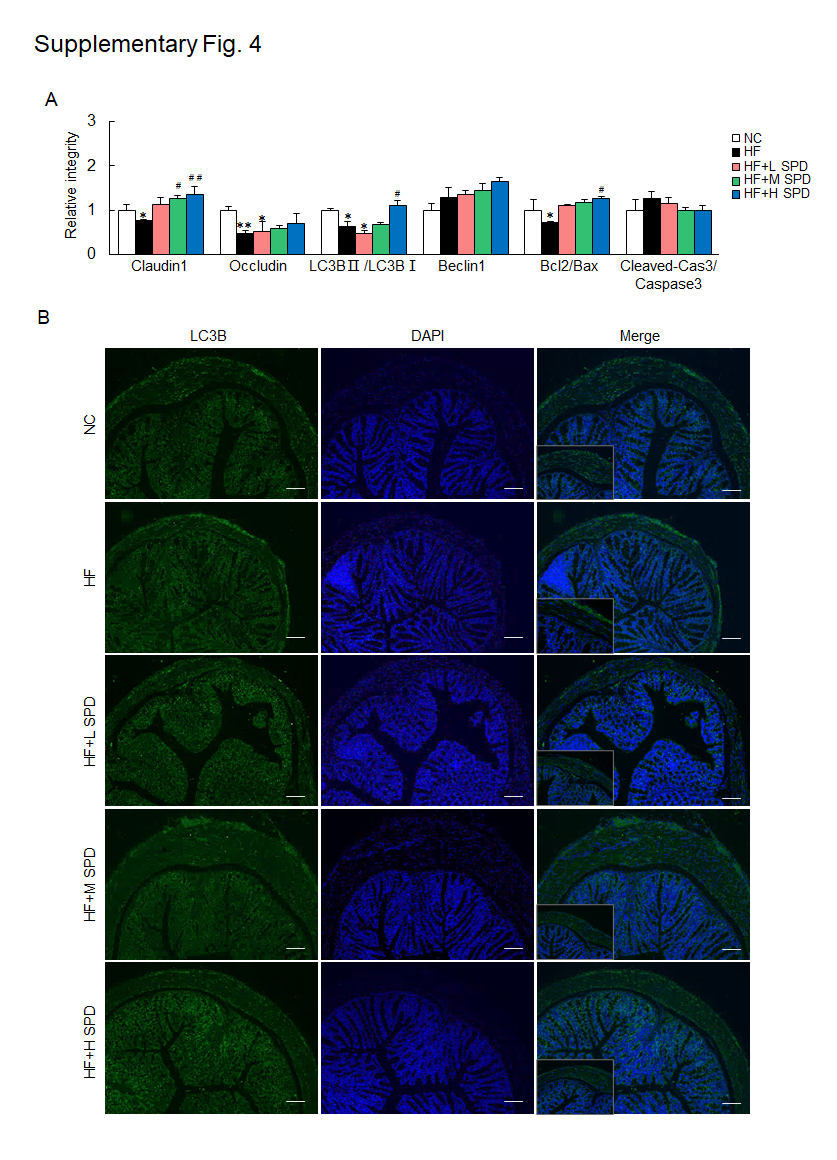

Supplement: Supplemental Material [file KGMI_A_1832857_SM6372.zip › Supplementary information/FigS4.tif]

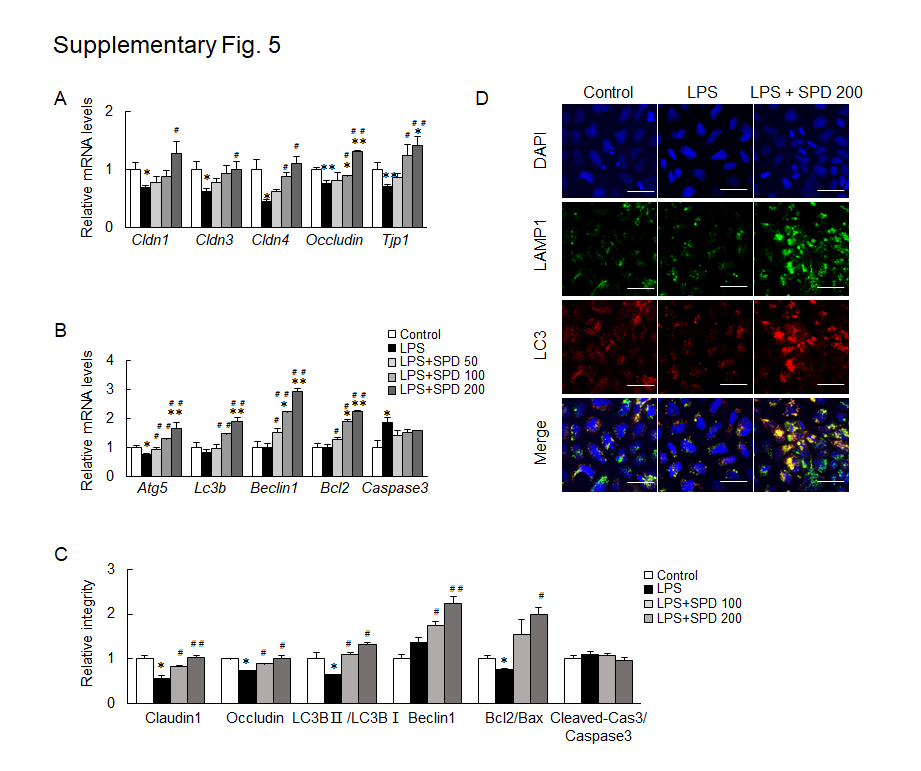

Supplement: Supplemental Material [file KGMI_A_1832857_SM6372.zip › Supplementary information/FigS5.tif]

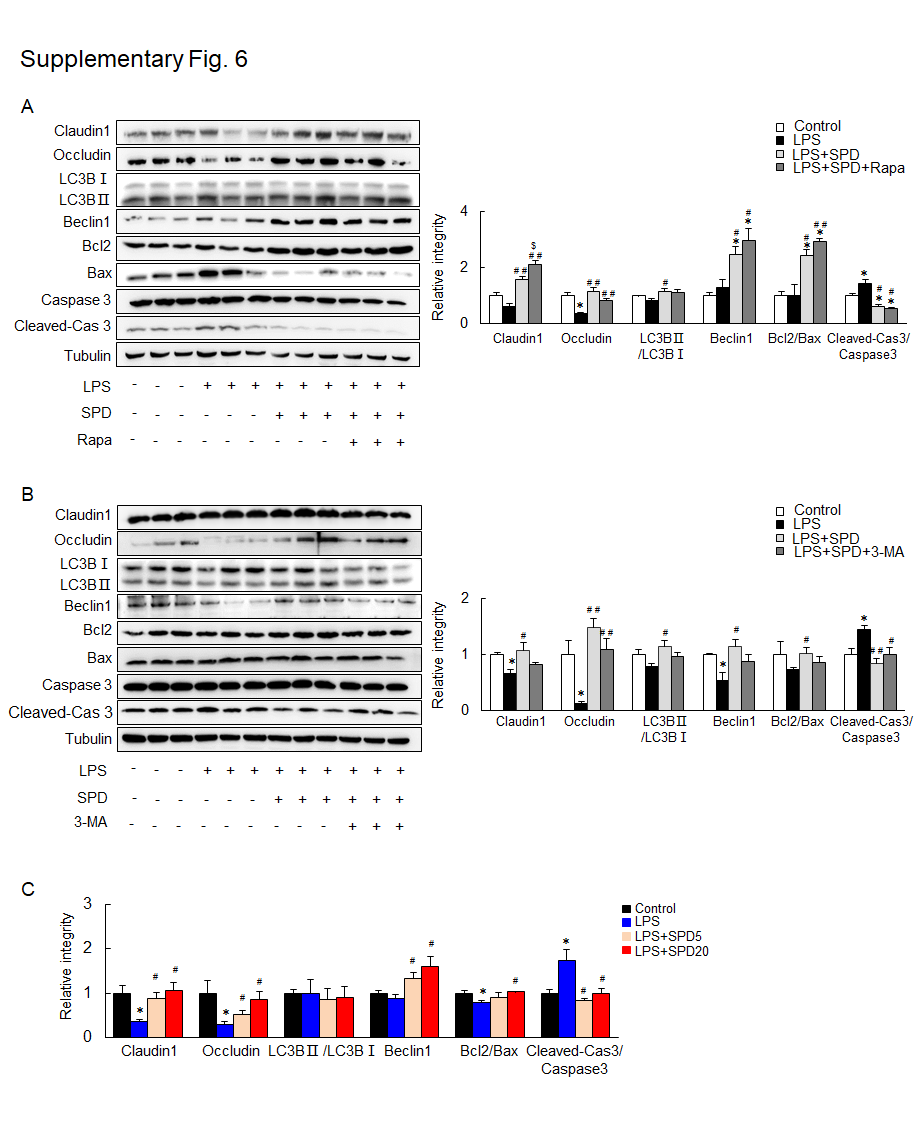

Supplement: Supplemental Material [file KGMI_A_1832857_SM6372.zip › Supplementary information/FigS6.tif]

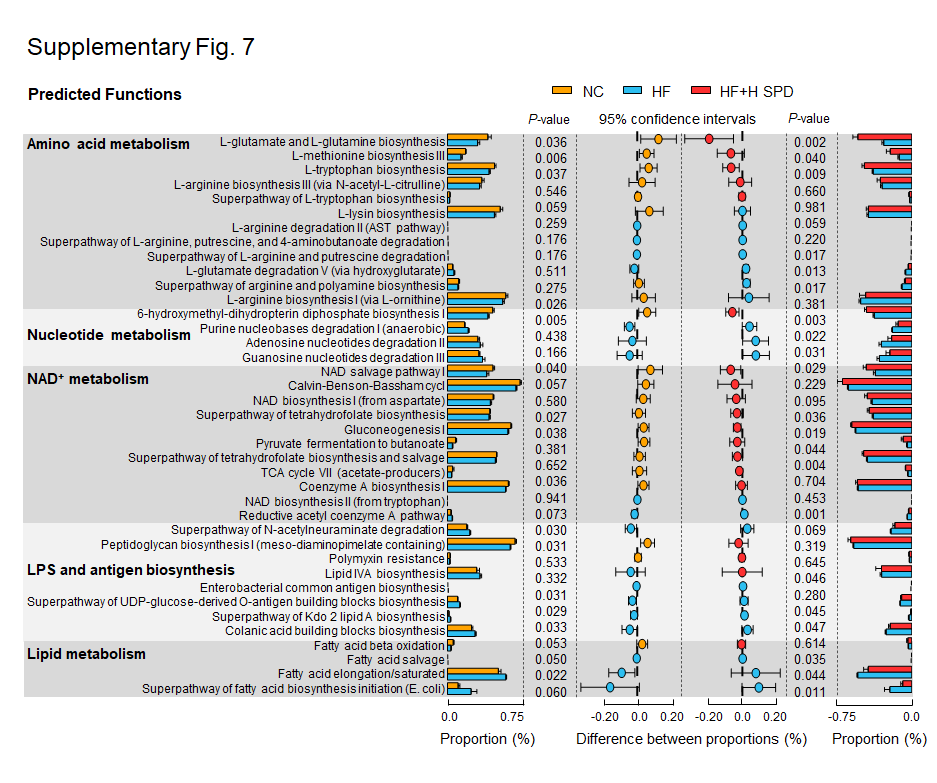

Supplement: Supplemental Material [file KGMI_A_1832857_SM6372.zip › Supplementary information/FigS7.tif]

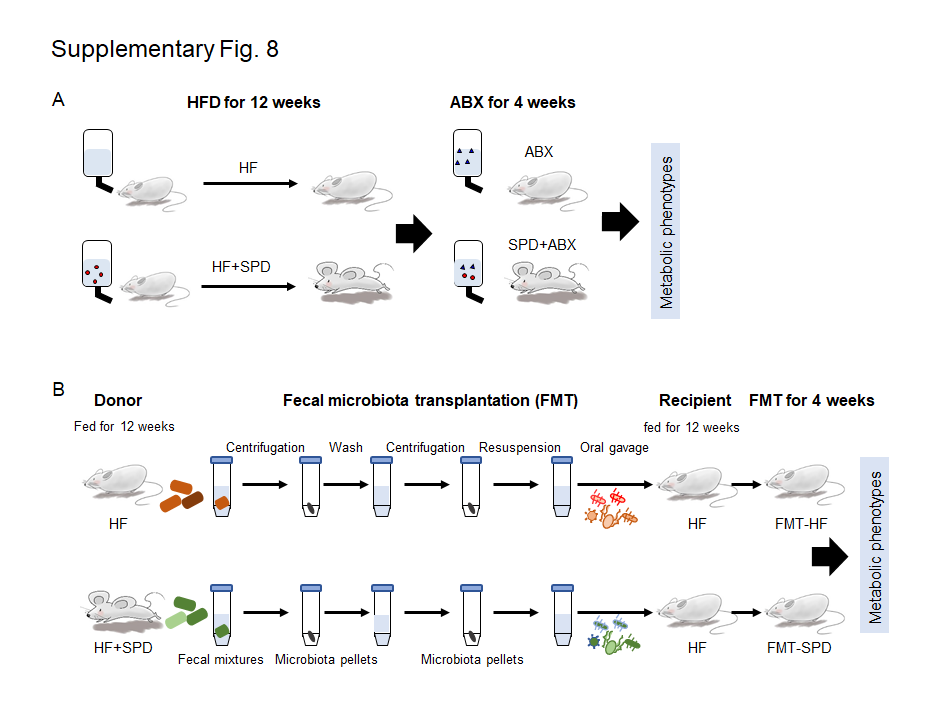

Supplement: Supplemental Material [file KGMI_A_1832857_SM6372.zip › Supplementary information/FigS8.tif]

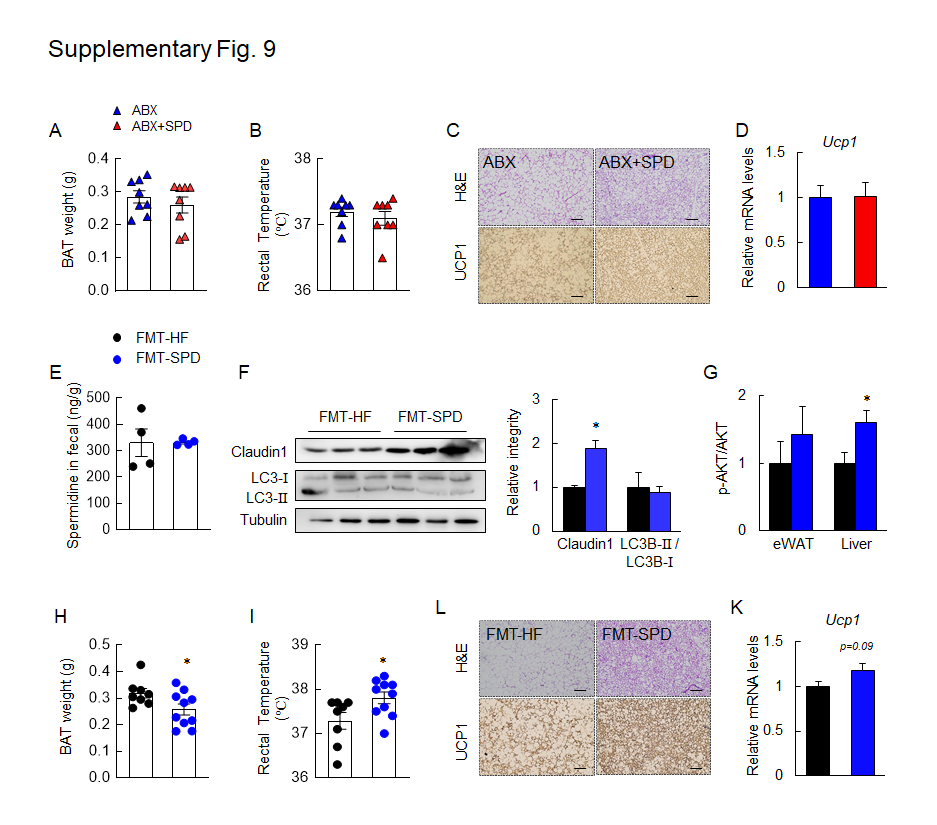

Supplement: Supplemental Material [file KGMI_A_1832857_SM6372.zip › Supplementary information/Figs9.tif]
